# Angiotensin receptor blockers modulate the lupus CD4^+^ T cell epigenome characterized by TNF family–linked signaling

**DOI:** 10.1172/jci.insight.176811

**Published:** 2024-12-17

**Authors:** Andrew P. Hart, Jonathan J. Kotzin, Steffan W. Schulz, Jonathan S. Dunham, Alison L. Keenan, Joshua F. Baker, Andrew D. Wells, Daniel P. Beiting, Terri M. Laufer

**Affiliations:** 1 Division of Rheumatology, Department of Medicine, and; 2 Institute for Immunology, Perelman School of Medicine, University of Pennsylvania, Philadelphia, Pennsylvania, USA.; 3 Division of Rheumatology, Department of Medicine, Corporal Michael J. Crescenz VA Medical Center, Philadelphia, Pennsylvania, USA.; 4 Center for Clinical Epidemiology and Biostatistics and; 5 Department of Pathology and Laboratory Medicine, Perelman School of Medicine, University of Pennsylvania, Philadelphia, Pennsylvania, USA.; 6 Department of Pathology, Children’s Hospital of Philadelphia, Philadelphia, Pennsylvania, USA.; 7 Department of Pathobiology, School of Veterinary Medicine, University of Pennsylvania, Philadelphia, Pennsylvania, USA.

**Keywords:** Autoimmunity, Immunology, Epigenetics, Lupus, T cells

## Abstract

In systemic lupus erythematosus (lupus), environmental effects acting within a permissive genetic background lead to autoimmune dysregulation. Dysfunction of CD4^+^ T cells contributes to pathology by providing help to autoreactive B and T cells, and CD4^+^ T cell dysfunction coincides with altered DNA methylation and histone modifications of select gene loci. However, chromatin accessibility states of distinct T cell subsets and mechanisms driving heterogeneous chromatin states across patients remain poorly understood. We defined the transcriptome and epigenome of multiple CD4^+^ T cell populations from patients with lupus and healthy individuals. Most patients with lupus, regardless of disease activity, had enhanced chromatin accessibility bearing hallmarks of inflammatory cytokine signals. Single-cell approaches revealed that chromatin changes extended to naive CD4^+^ T cells, uniformly affecting naive subpopulations. Transcriptional data and cellular and protein analyses suggested that the TNF family members, TNF-α, LIGHT, and TWEAK, were linked to observed molecular changes and the altered lupus chromatin state. However, we identified a patient subgroup prescribed angiotensin receptor blockers (ARBs), which lacked TNF-linked lupus chromatin accessibility features. These data raise questions about the role of lupus-associated chromatin changes in naive CD4^+^ T cell activation and differentiation and implicate ARBs in the regulation of disease-driven epigenetic states.

## Abstract 2

CD4+ T cells in lupus have TNF family-associated epigenetic changes that may be modulated by angiotensin receptor blockers

## Introduction

Systemic lupus erythematosus (SLE; lupus) is a model systemic autoimmune disease; environmental effects acting within a permissive genetic background result in the breakdown of immune tolerance. Dysfunction of multiple cell types is associated with anti-nuclear autoantibody (ANA) production and tissue pathology (1). Antigen-presenting cells are inappropriately activated, contributing to self-antigen presentation, cytokine-driven inflammation, and activation of adaptive immune cells (1, 2). The production of ANAs by B cell populations is a defining characteristic of disease (3). However, B cell–directed clinical therapies are imperfect, evidence suggests ANAs and tissue pathology are dependent on CD4^+^ T cells (Th cells) (4), and rituximab induces depletion of B cells. For example, the HLA class II locus is the strongest human genetic susceptibility allele, consistent with Th cell involvement in disease, and ANAs are high-affinity, class-switched antibodies that arise through germinal center (GC) responses dependent on follicular Th cells (Tfh cells) (5–8). Likewise, Th1 cells, Th17 cells, regulatory T cells (Tregs), and TCRαβ^+^CD4^–^CD8^–^ double-negative SLE T cells have abnormal intrinsic signaling, have altered frequencies, and invade tissues to contribute to pathology (7, 9–14).

Epigenetics has become an active area of study in lupus T cell biology, as genetic susceptibility alone poorly explains changes in Th subset frequencies and functions. Lupus Th cells have hypomethylation of DNA surrounding costimulatory genes (*CD40L*, *CD9*) (15, 16) and interferon (IFN) response genes (17, 18), as well as altered histone modifications at cytokine loci (*IL17*) (19). Lupus flare correlates with global changes in T cell DNA methylation (20). However, studies of T cell epigenetics in lupus have often relied on bulk Th cells containing multiple subsets of naive, activated, and memory populations (15, 16, 19, 21–24). Limited studies have distinguished naive Th cells from other populations (17, 18, 20), and prior naive Th isolation methods do not remove effector memory subsets (25, 26). Thus, epigenetic states of discrete Th populations including Tfh and naive subsets remain incompletely described.

The accessibility of chromatin regions across the genome varies in different cell types and is influenced by activation, cell cycling, cytokine signaling, and other processes leading to transcriptional responses (27). Direct measurement of chromatin accessibility by assay for transposase-accessible chromatin with sequencing (ATAC-Seq) has emerged as a tractable measure of epigenetic cell state and transcriptional potential. Prior studies of T cell chromatin accessibility in lupus have focused on peripheral blood mononuclear cells (PBMCs) without identifying individual T cell subsets (28), or Tregs (29), leaving unanswered questions about chromatin regulation in other T cell subsets.

We utilized ATAC-Seq to profile the chromatin accessibility of multiple discrete Th cell subsets — including naive Th and Tfh — implicated in lupus. All populations of lupus Th cells had a disease-specific chromatin signature. We verified that type I IFN transcriptional responses are present, but variable, in lupus Th cells; in parallel, the chromatin surrounding type I IFN–associated loci showed increased accessibility in lupus. However, Th cells had more penetrant increased accessibility at gene loci involved in other cytokine signaling pathways and leukocyte activation, including enrichment of accessibility at loci containing common transcription factor motifs (NF-κB, activator protein 1 [AP-1], interferon regulatory factor 1 [IRF1]). Although activation of type I IFN signaling has been associated with disease severity and autoantibody production, lupus-specific changes in Th chromatin accessibility were more tightly associated with transcription of loci related to TNF family signaling. Transcriptional activation of TNF signaling in lupus Th cells correlated with plasma levels of TNF family cytokines, including TWEAK, and with protein dysregulation of TNF family receptors, including HVEM (herpes virus entry mediator: TNFRSF14). Strikingly, lupus-associated epigenetic changes in T cells were absent in patients prescribed angiotensin receptor blocker (ARB) drugs, which have previously been shown to modulate NF-κB–dependent pathways. Instead, patients prescribed ARBs displayed heightened type I IFN responses unassociated with disease severity and potentially linked to downregulation of the TNF pathway. Thus, we find that the disease-specific epigenetic signature in Th cells in lupus is associated with TNF family members in addition to type I IFNs and may be modulated by ARBs.

## Results

### Lupus Th cells retain open chromatin features that define T cell subsets.

**Figure 1:**
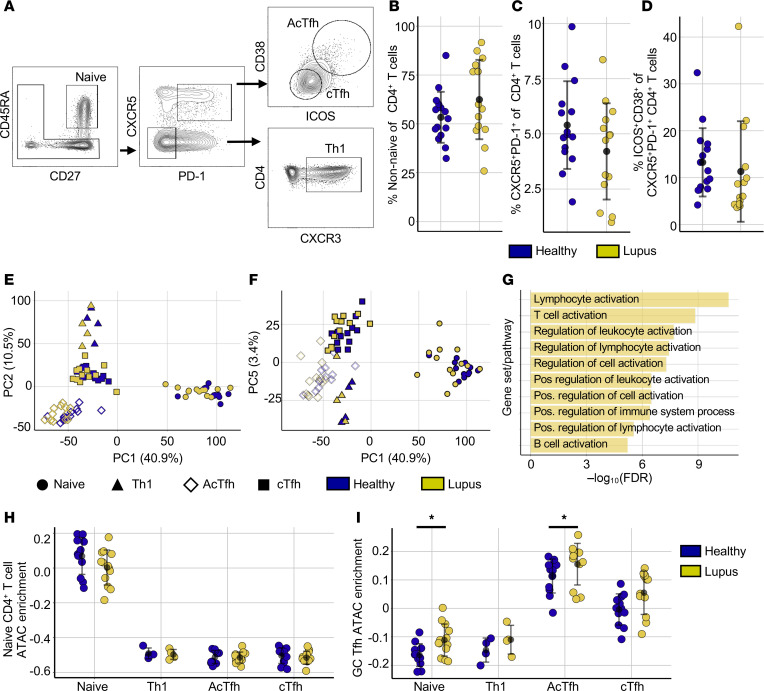
Lupus Th cells retain open chromatin features that define T cell subsets. (**A**) Representative gating strategy to purify CD45RA^+^CD27^+^ naive Th, CXCR5^+^PD1^+^ICOS^+^CD38^+^ AcTfh, CXCR5^+^PD1^+^ICOS^–^CD38^–^ cTfh, and CXCR5^–^PD1^–^CXCR3^+^ Th1 cells. PD1, programmed cell death 1; AcTfh, effector Tfh; cTfh, circulating Tfh. (**B**) Frequency of non-naive Th cells among CD4^+^ T cells in lupus (*n* = 14) and healthy individuals (*n* = 15) by flow cytometry. (**C**) Frequency of CXCR5^+^PD1^+^ T cells among CD4^+^ T cells in lupus (*n* = 14) and healthy individuals (*n* = 15). (**D**) Frequency of AcTfh cells among CD4^+^ T cells in lupus (*n* = 14) and healthy individuals (*n* = 15). (**E**) Principal component analysis (PCA) (PC1 × PC2) of ATAC-Seq data for sorted Th cell populations. (**F**) PCA (PC1 × PC5) of ATAC-Seq data for sorted Th cell populations. Colors distinguish lupus or healthy samples and shapes distinguish Th cell subsets. (**G**) The 10 most significant ChipEnrich pathway enrichment results for peaks defining non-naive CD4^+^ T cells in PC1. (**H**) Sample-wise peak-set variation analysis of ATAC-Seq data across lupus and healthy Th cell populations against published chromatin peaks enriched in naive Th cells. (**I**) Sample-wise peak-set variation analysis of published chromatin peaks enriched in GC Tfh cells across lupus and healthy Th cell populations. Error is reported as SD. ATAC-Seq data represent 25 naive Th cell samples (13 lupus, 12 healthy), 8 Th1 samples (4 lupus, 4 healthy), 24 cTfh samples (12 lupus, 12 healthy), and 24 AcTfh samples (12 lupus, 12 healthy). *P < 0.05, unpaired 2-tailed *t* tests (**B**–**D**, **H**, and **I**).

We sorted 4 distinct Th cell subsets to examine how the chromatin accessibility landscape is disrupted in lupus (Figure 1A). CXCR5^+^PD1^+^ (cTfh) cells are heterogeneous cells that include recently activated Th, memory Tfh, and AcTfh (CD38^+^ICOS^+^) cells (30). AcTfh cells are rare in circulation and more closely resemble GC Tfh cells transcriptionally and epigenetically (30). Changes in the biology of Tfh cells may contribute to pathology and ANA production in lupus. To determine if any changes observed were specific to Tfh differentiation, we also analyzed CXCR3^+^ circulating Th1 cells and CD45RA^+^CD27^+^ naive Th cells (Figure 1A).

ANA^+^ lupus individuals with low-to-moderate disease activity were recruited from outpatient clinics at the University of Pennsylvania. This cohort was largely female (92.3%), was predominantly of Black (54%) and White (38%) self-reported race, possessed a median SLE Disease Activity Index (SLEDAI) of 4 (range 0–12), had a median age of 38 years, and was treated with hydroxychloroquine (77%), mycophenolate mofetil (15%), corticosteroids (46%), and other drugs (Supplemental Table 1; supplemental material available online with this article; https://doi.org/10.1172/jci.insight.176811DS1). Anonymous healthy control (HC) samples were collected from routine donors to the Human Immunology Core at the University of Pennsylvania. Upon flow cytometry, PBMCs in a subset of patients had nonsignificant increases in non-naive Th cells (CD45RA^+^CD27^–^, CD45RA^–^CD27^–^, CD45RA^–^CD27^+^) (Figure 1B). There is no consensus on the dynamics of cTfh cells in lupus; in our cohort, cTfh cells were not expanded in lupus (Figure 1C) (29). There was also no significant difference in the percentage of circulating AcTfh cells between groups (Figure 1D).

Th cells were enriched from cryopreserved PBMCs, and 5,000 cells of each subset were sorted for low-input transcriptional and ATAC-Seq analyses (Figure 1A) (31). ATAC-Seq data were aligned to the genome, and chromatin accessibility peaks were identified using standardized Encyclopedia of DNA elements (ENCODE) pipeline methods (32, 33). Peaks were merged and filtered, and downstream analyses were performed using a consensus peak set containing 70,758 peaks integrated across all 4 cell subsets.

We first wanted to understand the depth of lupus epigenetic dysregulation. ATAC-Seq data were first applied to principal component analysis (PCA). Lupus Th cell subsets and their healthy counterparts clustered together in PC1, PC2, and PC5 (Figure 1, E and F). Representative accessibility tracks demonstrated comparable accessibility patterns in lupus and healthy cells at these loci (Supplemental Figure 1A). PC1, which contained 40.9% of the variation, divided naive from activated Th cell populations (Figure 1E). Chromatin peaks contributing to the clustering of non-naive cells along PC1 were annotated to genes and analyzed using pathway analysis: Enrichment results pointed to processes of immune activation including T cell activation (Figure 1G). To assay if lupus T cell subsets maintain established epigenetic identities during disease, we extracted ATAC-Seq peaks enriched in naive Th cells from published works and performed sample-wise peak set enrichment analysis (34). Naive Th cells of both lupus and healthy individuals were similarly enriched for the published naive chromatin peak set relative to activated cells (Figure 1H). We similarly examined Tfh identity utilizing published ATAC-Seq peaks enriched in GC Tfh cell populations (30). Lupus and HC AcTfh cells most closely resembled GC Tfh cells, with cTfh cells also showing moderate enrichment for GC Tfh peaks (Figure 1I). These data suggest that epigenetic cellular identity is maintained in lupus. Interestingly, naive Th and AcTfh cell populations in lupus were slightly more enriched for GC Tfh peaks compared with healthy cells (Figure 1I). Thus, although chromatin peaks defining separate subsets are maintained in lupus, disease-driven changes in accessibility are also apparent.

### The lupus epigenome is characterized by enhanced chromatin accessibility in regions surrounding T cell activation and cytokine signaling genes.

**Figure 2:**
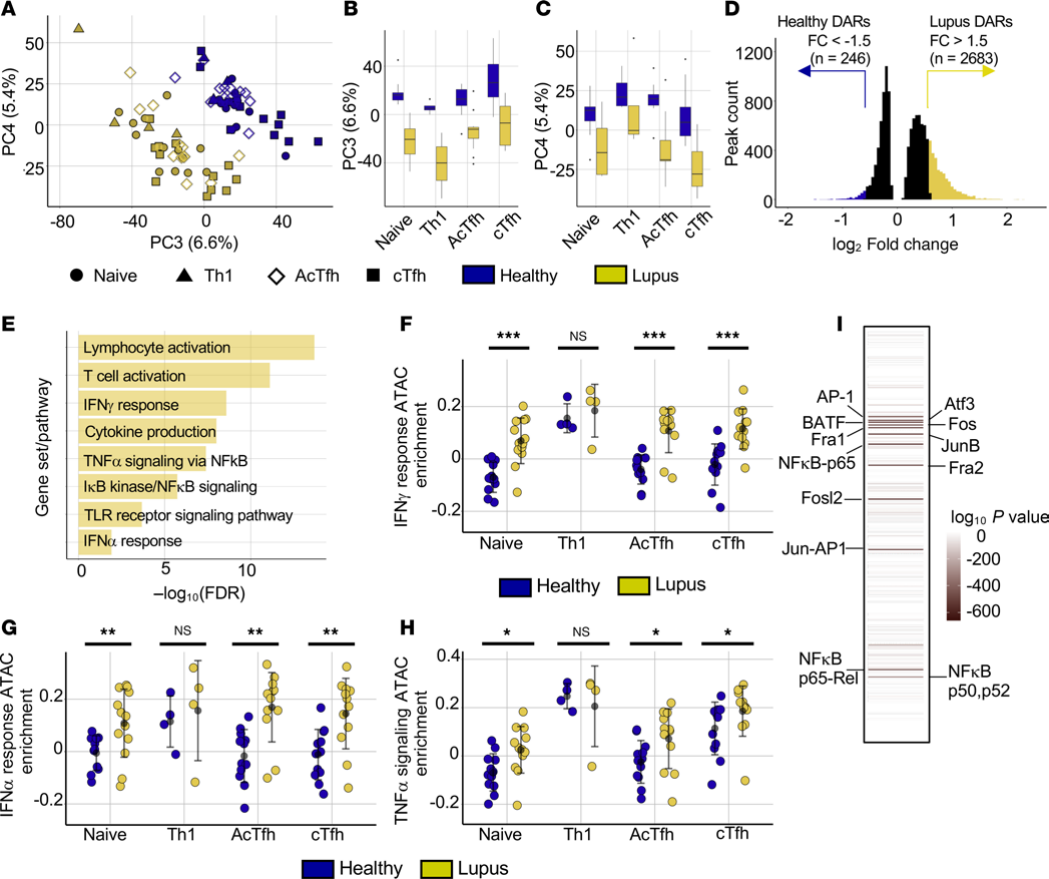
The lupus epigenome is characterized by enhanced chromatin accessibility surrounding T cell activation and cytokine signaling genes. (**A**) PCA plot (PC3 × PC4) of ATAC-Seq data for sorted Th populations. Colors distinguish lupus or healthy samples and shapes distinguish Th subsets. (**B** and **C**) PCA loadings separated by disease and cell type for PC3 (**B**) and PC4 (**C**). Box plots show the interquartile range, median (line), and minimum and maximum (whiskers). (**D**) Quantitation of differentially accessible regions (DARs) between lupus and healthy combined Th cells defined as adjusted *P* (padj) < 0.025 (*n* = 12,625) and depicted in black. Regions more accessible in lupus samples (*n* = 2,683) (padj < 0.025 & fold-change [FC] > 1.5) in yellow. Regions more accessible in HCs (*n* = 246) (padj < 0.025 & FC > 1.5) in blue. (**E**) Pathway enrichment analysis of lupus DARs (padj < 0.025 & FC > 1.5) (*n* = 2,683 regions) among GO Biological Process and MSigDB Hallmark gene sets. (**F**–**H**) Sample-wise peak-set variation enrichment scores for Hallmark IFN-γ response gene loci (**F**), Hallmark IFN-α response gene loci (**G**), and Hallmark TNF-α signaling gene loci (**H**). (**I**) HOMER TF motif analysis results and top TF motifs enriched in lupus DARs (*n* = 2,683). Error is reported as SD. ATAC-Seq data represent 25 naive Th samples (13 lupus, 12 healthy), 8 Th1 samples (4 lupus, 4 healthy), 24 cTfh samples (12 lupus, 12 healthy), and 24 AcTfh samples (12 lupus, 12 healthy). *P < 0.05, **P < 0.01, ***P < 0.001, unpaired 2-tailed *t* tests (**A**–**I**).

Chromatin accessibility patterns separated Th subsets and reiterate that Th cells circulating in participants with lupus are epigenetically similar to those in HCs (Figure 1E). However, additional PCs were lupus specific (Figure 2A). PC3 and PC4, representing 12% of total variation, separated lupus individuals from HCs. All Th cell subsets we examined were affected by lupus-driven PCs, indicating a shared set of chromatin changes (Figure 2, B and C). Using the combined data containing all Th cell subsets and controlling for cell subset–driven variation not altered by disease, we identified 12,625 chromatin peaks that were differentially accessible (differentially accessible regions, DARs) between lupus and healthy Th cells (FDR < 0.025). We restricted subsequent analyses to DARs exceeding a log_2_ fold-change of 0.58 (fold-change ~1.5). This revealed a bias toward opening of chromatin regions in lupus with increased accessibility in 2,683 regions in lupus T cells and only 246 regions of increased accessibility in HC T cells (Figure 2D and Supplemental Data 1).

Regions of accessible chromatin can prime transcription, recruit chromatin-modifying complexes, or act as enhancers of distal genes. We found that approximately 45% of all chromatin regions in the data were located within 3 kb of a gene promoter, but only 25% of the 2,683 lupus DARs were located in gene promoter regions (Supplemental Figure 2A) (35). In contrast, there was a relative enrichment of intronic regions making up nearly 50% of DARs (Supplemental Figure 2A). Interested in how Th chromatin accessibility might relate to lupus susceptibility SNPs, we identified linked (*r*^2^ > 0.5) proxy SNPs using published lupus SNP data and determined whether they overlapped with open chromatin in Th cells (8, 36). Among 6,103 proxy SNPs, 198 overlapped regions of open chromatin in the combined Th dataset: Roughly half of these occurred in gene promoter regions (Supplemental Figure 2B and Supplemental Data 2). Higher powered future analyses are needed to understand potential causal relationships between these susceptibility loci and accessibility and links between altered accessibility and 3D chromatin architecture.

To identify active transcriptional pathways involved in the observed differential accessibility of lupus Th cells, we utilized gene sets and pathways defined in Gene Ontology Biological Processes (37) and Molecular Signatures Database (MSigDB) Hallmark gene sets (38). DARs showing increased accessibility in lupus were enriched for programs including lymphocyte activation, T cell activation, and multiple Hallmark cytokine signaling pathways, including those of IFN-γ, TNF-α, and IFN-α (Figure 2E). IFN-γ and IFN-α response pathways were enriched in multiple lupus Th cell subsets, including naive Th and Tfh populations (Figure 2, F and G). Fewer Th1 samples were isolated and analyzed, and they were not significantly enriched for these pathways. Genes involved in the TNF-α signaling pathway were enriched among lupus DARs in all subsets except Th1-like cells, which had high baseline enrichment in HCs (Figure 2H). We next asked if DARs were localized over known DNA-binding motifs of transcription factors (TFs) associated with regulating these pathways. Motifs binding AP-1 family and NF-κB family TFs appeared among the most highly enriched, as might be expected downstream of cytokines such as TNF-α (Figure 2I). IRF3 and IRF1 motifs, downstream of interferon signaling, were also enriched in this dataset (Supplemental Data 3). Motif enrichment analysis limited to lupus DARs located in gene promoters identified nearly identical motif enrichments (Supplemental Figure 2C).

All Th cell subsets from lupus participants clustered together in PC3 and PC4 (Figure 2B), and therefore there is an epigenetic signature specific to lupus and independent of cell subset (Figure 2, E–G). However, we also asked if there were disease-linked chromatin changes specific to particular Th cell subsets. We performed pairwise comparisons of lupus and healthy naive, cTfh, and AcTfh cells. For each comparison, we identified DARs (FDR < 0.025) separating lupus and healthy cells, and we examined whether DARs with increased accessibility in lupus overlapped across evaluated T cell subsets. We observed increased accessibility in 1,141, 958, and 1,539 DARs in naive, AcTfh, and cTfh lupus cells, respectively. More than 50% of DARs identified in each subset were also identified in at least 1 other subset, indicating substantial overlap (Supplemental Figure 2D). In contrast, there were 493, 329, and 709 chromatin peaks in naive, AcTfh, and cTfh cells, respectively, which we identified as differentially accessible (FDR < 0.025) in only 1 lupus Th cell subset (Supplemental Figure 2D). Lupus DARs specific to AcTfh and cTfh cells did not show significant (FDR < 0.05) pathway enrichments. Naive Th cell–specific DARs (*n* = 493) were significantly enriched for Hallmark inflammatory response and Hallmark TNF-α signaling gene sets (Supplemental Data 4). Many subset-specific lupus DARs in different T cell subsets surrounded the same gene loci. For example, different peaks in the ICOS locus showed increased accessibility in lupus AcTfh, naive, and cTfh cells (Supplemental Figure 2E). Cell subset–specific accessibility, transcriptomes, and TF activity might regulate lupus responses, but these responses activate similar gene networks and pathways across different cell subsets.

### Combined transcriptional and chromatin accessibility data highlight cytokine-driven dysregulation in lupus.

**Figure 3:**
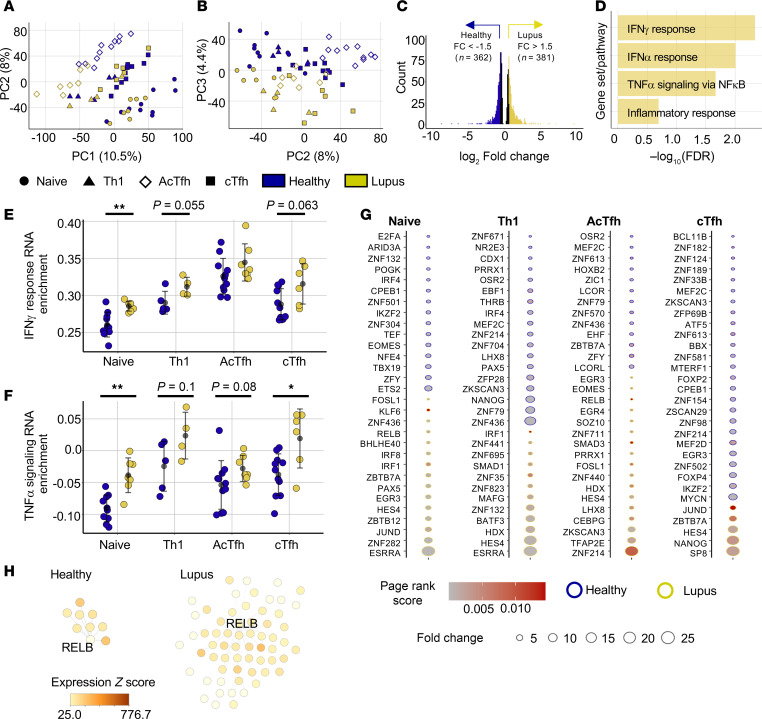
Transcriptional and chromatin accessibility data highlight cytokine-driven dysregulation in lupus. (**A** and **B**) PCA (PC1 × PC2 in **A** and PC2 × PC3 in **B**) of RNA-Seq data for Th subsets. Colors indicate lupus or healthy samples and shapes indicate Th subsets. (**C**) RNA-Seq–defined differentially expressed genes (DEGs) between lupus and healthy samples (naive, AcTfh, cTfh, Th1) defined as padj < 0.05 (black) (*n* = 1,132). Genes with higher expression in lupus samples (*n* = 381) (padj < 0.05 & FC > 1.5) in yellow. Genes with higher expression in healthy samples (*n* = 362) (padj < 0.05 & FC > 1.5) in blue. (**D**) Gene set enrichment analysis (GSEA) pathway results for lupus DEGs (*n* = 381) among Gene Ontology (GO) Biological Process and MSigDB Hallmark gene sets. (**E** and **F**) Gene set variation analysis (GSVA) enrichment of Hallmark IFN-γ response gene set (**E**) and Hallmark TNF-α signaling via NF-κB gene set (**F**) in Th populations of lupus participants and HCs. (**G**) Differentially active TFs in lupus and healthy Th subsets with a Taiji page rank score > 0.0002 in either lupus or healthy populations. The 30 TFs with the highest FC (indicated by circle size) between lupus and healthy conditions for each cell type are displayed; greater TF page rank activity score in healthy participants (blue) or lupus participants (yellow). (**H**) Taiji-defined RELB TF gene regulatory networks among lupus or healthy naive Th cells (edge weight cutoff = 100). Node color saturation is proportional to node expression. Error is reported as SD. RNA-Seq data represent 17 naive Th samples (7 lupus, 10 healthy), 9 Th1 samples (4 lupus, 5 healthy), 17 cTfh samples (6 lupus, 11 healthy), and 18 AcTfh samples (7 lupus, 11 healthy). *P < 0.05, **P < 0.01, paired 2-tailed *t* tests (**A**–**H**).

Lupus Th cells are inappropriately activated by self-antigen, immune complexes, and cell-cell interactions. Transcriptional studies have highlighted type I IFN responses in lupus Th cells and PBMCs (39–41). We demonstrated enhanced chromatin accessibility surrounding genes involved in lymphocyte activation, TNF-α signaling, and IFN-α and IFN-γ responses in lupus Th cells (Figure 2). We utilized RNA-Seq transcriptional profiling of the same samples to assay if epigenetic changes correlated with transcriptional activity. There was separation of distinct Th cell subsets in PCA (Figure 3A). Similar to epigenetic data, lupus-specific transcription was present in all subsets (Figure 3B). After integration of the samples, more than 700 genes were differentially expressed between lupus and HCs (padj < 0.05, FC > 1.5) (Figure 3C). Many genes contained in the Hallmark IFN-α response and Hallmark TNF-α signaling pathways, including *IFI27*, *IFI44*, *RELB*, *KLF6*, and *CD83*, had significantly higher expression in lupus: We verified transcriptional changes in the naive compartment by quantitative PCR (qPCR) (Supplemental Figure 3A). Gene set enrichment analysis (GSEA) identified enrichment of these pathways and the IFN-γ response pathway in most cell populations (Figure 3D). Gene set variation analysis (GSVA) was used to measure enrichment of these pathways in individual lupus participants and cell subsets, and IFN-γ, TNF-α, and IFN-α pathways were transcriptionally enriched in lupus naive, AcTfh, and cTfh cell subsets (Figure 3, E and F, and Supplemental Figure 3B). The enrichment of IFN-γ and TNF-α pathways was not significant in Th1 cells.

We leveraged these data containing matched chromatin accessibility and transcriptional profiles to define which TFs drive the lupus phenotype in each Th subset. Taiji integrates chromatin accessibility at TF motifs and expression of TFs with expression of target genes to generate a page rank score, a quantitative measure of TF activity (42). We used Taiji to identify differentially active TFs in lupus and healthy Th cells and point to potential drivers of dysregulation (Figure 3G). TFs with the highest activity (page rank score > 0.002) were selected, and then among those TFs, TFs with the greatest FC in activity between lupus and healthy conditions were examined. The results highlighted IFN activity: IRF8 and IRF1 appeared differentially active in naive and Th1 cells (Figure 3G). However, a much larger set of TFs contained in the TNF-α signaling gene set appeared among the active TFs in lupus cells; these include FOSL1, KLF6, RELB, BHLHE40, EGR3, and SMAD3 (Figure 3G). Visualized differently, the networks of regulated gene targets of RELB, KLF6, and IRF1 in naive Th cells were much larger than those of healthy Th cells (Figure 3H and Supplemental Figure 3, C and D). Thus, there is a robust difference in regulatory activity of these TFs in disease, and these data corroborate our findings to implicate aberrant cytokine stimulation across multiple Th cell populations. These data also suggest that IFN-α, a canonical lupus-associated cytokine, does not act alone to alter cell profiles.

### Single-cell multiome analysis of naive Th cells demonstrates ubiquitous dysregulation in lupus.

We have shown that lupus naive Th cells retain the characteristics of naive Th cells and cluster with healthy naive cells (Figure 1E). However, these cells also have lupus-specific epigenetic and transcriptional signatures linked to activation and cytokine signaling. Expansion of a novel CD45RA^+^CD27^+^ antigen-experienced population among lupus naive T cells or shifts in the frequencies of naive Th cell subpopulations could explain this result and potentially indicate that quiescent true naive T cells are unaltered in disease. Alternatively, genetic susceptibility to lupus or direct influence of circulating signals, including cytokines, may lead to chromatin opening preceding T cell activation and affecting naive resting populations.

**Figure 4:**
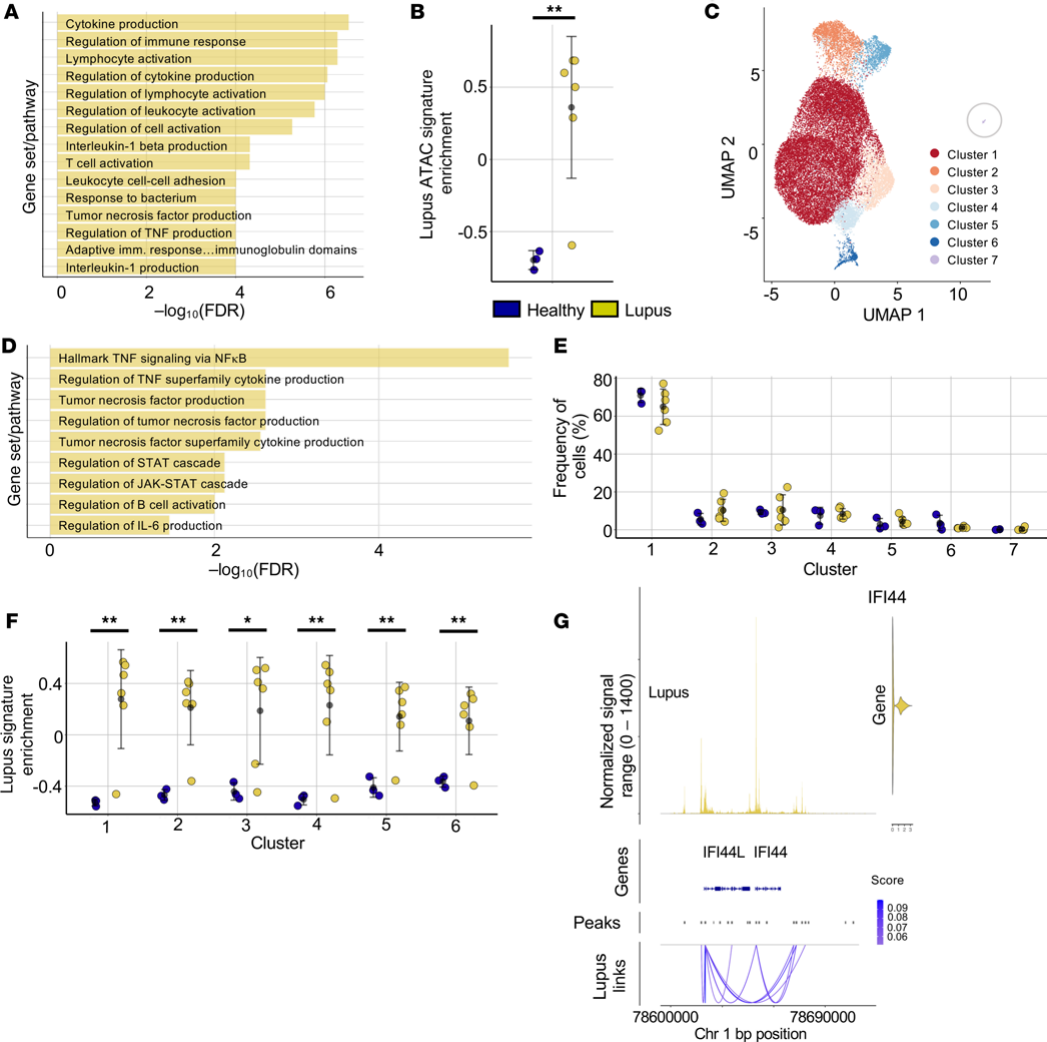
Single-cell multiome analysis of naive Th cells demonstrates ubiquitous dysregulation in lupus. (**A**) Pathway enrichment analysis results for 10x Genomics single-cell ATAC–defined (scATAC-defined) DARs of greater accessibility in lupus naive Th cells. Pathways used in enrichment include GO Biological Process and Hallmark gene sets. (**B**) GSVA enrichment score for lupus-associated DARs (Figure 2C, n = 2,683 regions) among integrated scATAC datasets of patients with lupus and HCs. (**C**) Uniform manifold approximation and projection (UMAP) dimensional reduction of multiomic single-cell clusters from naive Th cells. Single-cell nuclear RNA and scATAC data from naive Th of lupus and healthy individuals were independently integrated and then combined for cluster analysis and UMAP visualization. (**D**) Pathway enrichment analysis results for scATAC-defined DARs of cluster 5 in naive Th cells. (**E**) Frequency of cells among naive Th clusters for individual lupus and healthy participants. (**F**) Enrichment of signature lupus-associated DARs (Figure 2C, n = 2,683) among naive Th clusters. (**G**) Peak-gene linkage analysis results depicting chromatin accessibility track (top), gene expression (top right), and peak-gene linkages (bottom) along the *IFI44* locus in lupus naive Th cells. Error is reported as SD. Single-cell multiome data include 6 lupus and 3 healthy participants (**A**–**G**). *P < 0.05, **P < 0.01, paired 2-tailed *t* tests (**B**, **E**, and **F**).

To examine these alternative hypotheses, we generated single-cell multiome chromatin accessibility and nuclear RNA data from sorted CD45RA^+^CD27^+^CD4^+^ T cells in 3 healthy and 6 lupus participants. We identified 237 DARs; 233 were more accessible in lupus, mirroring the enhanced accessibility found in bulk ATAC-Seq results. The 233 lupus DARs demonstrated significant enrichment of expected pathways, including cytokine production and tumor necrosis factor production (Figure 4A). We next directly compared the bulk and single-cell data sets by asking whether the DARs identified in bulk ATAC-Seq analyses were enriched in single-cell chromatin profiles of lupus participants. The peak set containing bulk ATAC-Seq–defined lupus DARs (Figure 2C) was highly enriched relative to HCs in all but 1 lupus sample (Figure 4B). Nuclear RNA data also demonstrated increased expression of many transcripts identified in bulk RNA-Seq assays, including *IFI44*, *KLF6*, *REL*, and *NFKB1*: genes contained in IFN-α– and TNF-α–related gene sets (Supplemental Data 5).

Single-cell nuclear RNA and ATAC-Seq data of sorted naive Th cells were independently integrated and merged and cells were clustered (43, 44). Six primary clusters containing unique chromatin accessibility peaks or gene expression patterns were identified (Figure 4C); 1 additional cluster (cluster 7) was present at very low frequency in 2 participants (1 healthy and 1 lupus). These clusters fit into established naive subpopulations in the literature. Cluster 2 had unique upregulation of *IL2RA* expression (Supplemental Figure 4A). Previous studies have described IL2RA^hi^ naive subpopulations as effector precursors or Treg-like naive cells (45, 46). Supporting this, chromatin surrounding *FOXP3* was more accessible in cluster 2 and cluster 5, and DARs defining cluster 2 were significantly enriched for the Hallmark IL2/STAT5 signaling pathway, which may relate to high expression of *IL2RA* (data not shown). Cells in cluster 5 had open chromatin around the *IFNG* locus and increased expression of *CCL5* and *GZMA* and are, therefore, similar to previously described stem cell memory-like CD45RA^+^CD27^+^CD4^+^ T cell populations (Supplemental Figure 4, B–D) (46, 47). The remaining 4 clusters have been labeled “true naive” populations in published analyses and are more tightly clustered along UMAPs (46). Among the 4 true naive clusters, cluster 3 exhibited the most significant differences, including increased *SOX4* expression (Supplemental Figure 4, E and F). Previous work has suggested that *SOX4*-expressing naive Th cell subpopulations are enriched in young individuals and associated with CXCL13 expression upon activation (46, 48). Thus, single-cell data recapitulate the lupus chromatin phenotype and prior studies of the naive Th cell compartment.

We asked whether specific clusters contributed more to the lupus phenotype. DARs defining cluster 5 were enriched for multiple TNF-related gene sets and JAK/STAT signaling (Figure 4D). Potential overabundance of this cluster might explain the lupus naive Th cell phenotype. However, no consistent or significant lupus-associated differences in naive cluster frequencies appeared (Figure 4E); thus, altered dynamics within subpopulations did not explain the lupus epigenetic phenotype in naive Th cells. To more accurately place where the chromatin changes identified in bulk ATAC-Seq occurred across the naive clusters, we used cluster-wise pseudobulk analyses to generate a lupus chromatin enrichment score for each sample. Lupus cells of every cluster were enriched for the lupus chromatin signature relative to healthy cells in the same cluster (Figure 4F). Similarly, multiple lupus DEGs had increased expression in lupus naive Th cells across all clusters (Supplemental Figure 4G). These data suggest lupus is not characterized by shifting frequencies of naive subpopulations, nor is the lupus signature restricted to one aberrant naive Th cell cluster. Rather, differential chromatin accessibility in lupus results from widespread changes to accessibility within all subpopulations of CD45RA^+^CD27^+^CD4^+^ T cells, including true naive Th cells.

Single-cell multiome analysis permits the direct association of chromatin accessibility with transcription (49). We performed peak-gene linkage analysis, identifying correlations between chromatin accessibility and gene expression for individual genes at the single-cell level. We examined peak-gene linkages for the 663 DEGs identified in nuclear RNA data and identified 637 peak-gene associations unique to lupus cells while 255 peak-gene associations were unique to healthy cells (Supplemental Data 6). Unique peak-gene linkages of lupus cells demonstrated an association of altered chromatin with differential gene expression at the single-cell level (Supplemental Data 6). An example of this is the *IFI44* locus, which has many peak-gene linkages in lupus cells (Figure 4G) and not in healthy cells (Supplemental Figure 4H).

Collectively, we find that disease-specific cellular heterogeneity does not drive the lupus signatures of naive Th cells. Existing heterogeneity among flow cytometry–defined CD45RA^+^CD27^+^ Th cells is maintained in lupus. Instead, lupus chromatin and transcriptional changes are widespread across naive subpopulations and cell states, consistent with a ubiquitous feature including environmental stimuli like cytokines or genetic polymorphisms affecting the naive Th cell pool broadly. Further, these data suggest a direct association between lupus-associated changes in accessibility and gene expression in naive Th cells.

### Lupus Th cells appear in 2 epigenetically distinct groups defined by transcriptional activation of TNF-α signaling pathway genes.

**Figure 5:**
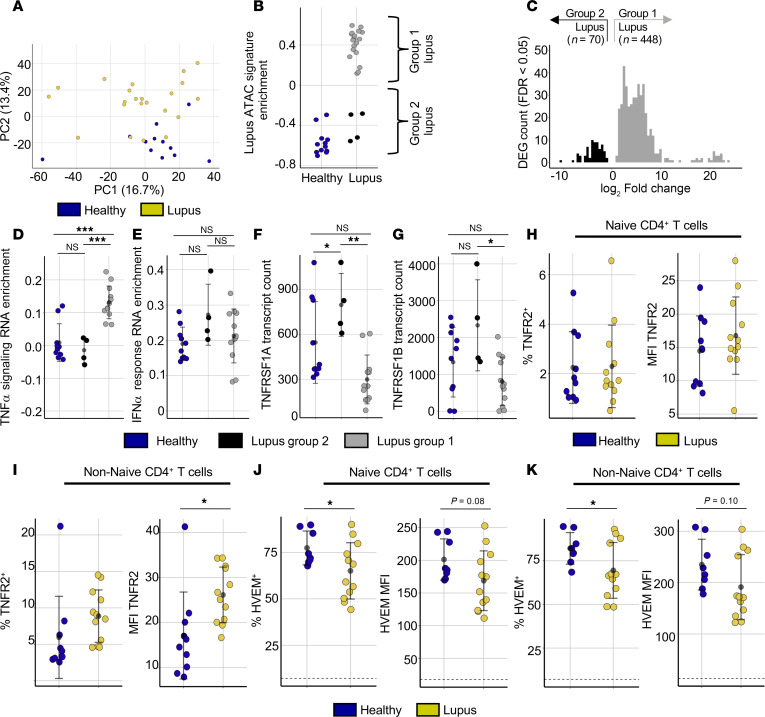
Lupus naive Th cells with open chromatin have a TNF signature. (**A**) PCA (PC1 × PC2) of ATAC-Seq data for naive Th cells from lupus and healthy individuals. Colors indicate lupus or healthy states. (**B**) Enrichment of signature lupus-associated DARs (Figure 2C, n = 2,683) among naive Th samples. Brackets highlight lupus samples grouped into either group 1 or group 2 based on lupus enrichment score. (**C**) DEGs from RNA-Seq data (padj < 0.05) between group 1 and group 2 lupus naive Th cells (**B**). Genes more highly expressed in group 1 (gray) or more highly expressed in group 2 (black) are indicated. (**D** and **E**) RNA-Seq data GSVA of Hallmark TNF-α signaling via NF-κB (**D**) and Hallmark IFN-α response gene sets (**E**) in naive Th cells in group 1 lupus participants, group 2 lupus participants, and HCs. (**F** and **G**) *TNFRSF1A* (**F**) and *TNFRSF1B* (**G**) gene transcript counts in group 1 lupus participants, group 2 lupus participants, and HCs. (**H**–**K**) Flow cytometry of lupus and HC PBMCs. (**H** and **I**) Frequency of TNFR2^+^ (right) and TNFR2 MFI (left) among naive (**H**) and non-naive (**I**) Th in patients with lupus (*n* = 12) and HCs (*n* = 10). (**J** and **K**) Frequency of HVEM+ (right) and HVEM MFI among naive (**J**) and non-naive (**K**) Th in patients with lupus (*n* = 12) and HCs (*n* = 8). Error is reported as SD. ATAC-Seq data represent 34 naive Th samples (22 lupus, 12 healthy) (**A** and **B**). RNA-Seq data represent 25 samples (16 lupus, 10 healthy) (**C**–**G**). 10x Genomics single-cell multiome data include 6 lupus and 3 healthy participants (**I**). *P < 0.05, **P < 0.01, ***P < 0.001, multiple 1-way ANOVA with Tukey’s multiple comparisons correction (**D**–**G**). *P < 0.05, **P < 0.01, ***P < 0.001, 2-tailed *t* tests (**H**–**K**).

Single-cell data reinforced the observation that naive Th cells in patients with lupus are epigenetically and transcriptionally altered. A small number of patients with lupus did not have the disease-specific epigenetic signature but segregated with healthy participants (Figure 2, F–H, and Figure 4B). To probe this variability, we added naive Th cells from additional participants. The expanded cohort had the expected increased accessibility of cytokine-driven pathways (Supplemental Figure 5A). In this larger cross-sectional analysis, 4 lupus samples clustered with healthy samples in PCA (Figure 5A). This effect became more apparent when using GSVA to assign a single value to the cumulative enrichment of lupus DARs in each sample (Figure 5B). Patients with lupus separated into 2 groups: Group 1 patients had modified chromatin accessibility of naive Th cells with the lupus signature while the epigenetic landscape of group 2 patients overlapped that of HCs (Figure 5B). Applying the same enrichment tests to cTfh, AcTfh, and Th1 cells, we found that group 2 lupus individuals with “healthy” naive Th cell chromatin also lacked lupus-specific chromatin dysregulation in activated populations (Supplemental Figure 5, B–D). The separation of patients with lupus into distinct groups explained variability in lupus patients seen at the single-cell level (Figure 4B). Thus, the dysregulation of chromatin in Th cells affects different cell subsets similarly but is found only within a subgroup of patients.

Clinical disease features and molecular profiles of patients with lupus have previously been used to identify subgroups within patients with lupus (40, 41). We asked if the 2 distinct lupus epigenetic subgroups correlated with molecular features. We compared the naive Th transcriptional profiles of the 2 lupus groups and identified 518 DEGs between the 2 groups, including 448 genes with elevated expression in patients with altered chromatin accessibility (group 1) (Figure 5C) and 70 genes with higher expression in group 2 lupus individuals (Supplemental Data 7). Our previous results demonstrated that lupus Th cells are enriched for cytokine signaling pathways including TNF-α signaling, IFN-α response, and IFN-γ response gene sets and we focused on these genes. We identified robust transcriptional enrichment of the TNF-α signaling pathway in group 1 patients with lupus and not in either group 2 patients with lupus or HCs (Figure 5D). Type I IFN responses, however, showed overlap between samples in the 2 lupus groups and variability among individuals (Figure 5E), and the IFN-γ response did not show bias between the lupus groups (Supplemental Figure 5E). We next asked if peak-gene linkages in the single-cell multiome dataset reflected the utilization of TNF-related genes. Among the 637 peak-gene linkages unique to lupus, 78 ATAC-Seq peaks were linked to 29 genes in the TNF-α signaling pathway, including *TNFAIP3* (Supplemental Figure 5, F and G, and Supplemental Data 6). These data corroborate TNF family cytokines as regulators of the lupus chromatin landscape and the existence of 2 epigenetically distinct lupus T cell phenotypes.

Increased expression of many TNF-α signaling genes, including *RELB*, accompanied the TNF-related enrichment in group 1 lupus participants. We examined gene expression of TNF family receptors in lupus groups and controls. *TNFRSF1A* (TNFR1) expression was significantly higher in group 2 lupus participants compared with group 1, and *TNFRSF1B* (TNFR2) followed a similar insignificant trend (Figure 5, F and G). Dysregulation of these receptors might indicate differential receptor utilization or feedback signaling. Other TNF family receptors, including TNFRSF12A (TWEAKR) and TNFRSF14 (HVEM), showed heterogeneous expression patterns but were not significantly altered between lupus groups (Supplemental Figure 5, H and I). The expression of the TNF family ligands for these receptors has previously been reported to change during lupus, including increased TNF-α concentrations in some patients (50, 51). TWEAK, the ligand of TNFRSF12A, has previously been reported to be involved in lupus pathology (52), and LIGHT (*TNFSF14*), a ligand of HVEM (*TNFRSF14*), is commonly dysregulated in murine lupus (53, 54).

We used flow cytometry of distinct cohorts to determine if TNF family receptors are dysregulated in lupus Th cells. TNFR1, TNFR2, and TWEAKR and high expression of HVEM were detected (Supplemental Figure 6A) in naive Th cells. Naive Th cells from lupus and healthy participants had similar MFI and percentage positive for both TNFR1 and TNFR2 (Figure 5H and Supplemental Figure 6, B and C). Non-naive Th cells did not show significant change in TNFR1 expression in patients with lupus (Supplemental Figure 6, D and E). In contrast, the MFI of TNFR2 in non-naive Th and cTfh cells was significantly increased in patients with lupus (Figure 5I and Supplemental Figure 6, F and G). TWEAKR expression was not different in lupus T cells (Supplemental Figure 6, H and I). The frequency of HVEM^+^ cells was reduced in both naive and non-naive lupus Th cells (Figure 5, J and K). HVEM expression was also reduced in CD8^+^ T cells and CD14^+^ monocytes from patients with lupus (Supplemental Figure 6, J and K). BTLA, the inhibitory binding partner of HVEM, was maintained at normal levels in lupus T cells (data not shown). In participants with concordant samples, we verified that dysregulated expression of HVEM correlated with the presence of the group 1 lupus epigenetic signature (Supplemental Figure 6, L and M). In these group 1 lupus individuals, there was reduced CD28 expression on Th cells, an effect that has been linked to TNF-α signaling (Supplemental Figure 6, N–P) (55, 56). Thus, dysregulated expression of both HVEM and TNFR2 and the association of the lupus epigenetic signature with transcriptional activation of TNF-α signaling suggest a complex environment in which multiple cytokines, potentially TNF-α or LIGHT, affect the molecular biology of Th cells.

### Prescriptions for ARBs correlate with naive Th cell epigenetic and transcriptional states in lupus.

**Figure 6:**
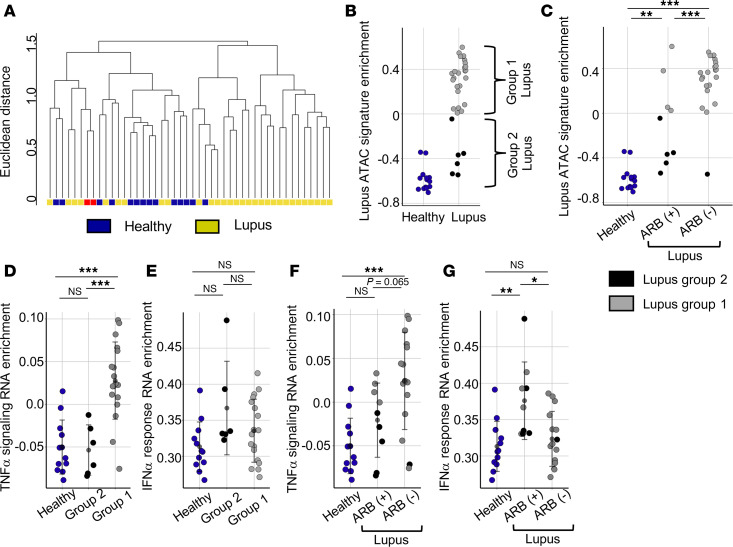
ARB prescription is associated with epigenetic and transcriptional changes in lupus T cells. (**A**) Dendrogram representing hierarchically clustered ATAC-Seq profiles of lupus and healthy samples. A single group 2 lupus participant appears twice and was tracked over more than 2 years and resampled (red). (**B**) Enrichment of signature lupus-associated DARs (Figure 2C, n = 2,683) among naive Th samples in this expanded dataset. Brackets highlight lupus samples grouped into either group 1 (gray) or group 2 (black) based on lupus enrichment score. (**C**) Enrichment of signature lupus-associated DARs (Figure 2C, n = 2,683) in naive Th cells graphed in healthy individuals and lupus individuals prescribed (ARB^+^) and not prescribed (ARB^–^) angiotensin receptor blocking drugs. (**D** and **E**) RNA-Seq data GSVA of Hallmark TNF-α signaling via NF-κB (**D**) and Hallmark IFN-α response gene sets (**E**) in naive Th cells in group 1 (gray) lupus participants, group 2 (black) lupus participants, and HCs (blue). (**F** and **G**) RNA-Seq data GSVA of Hallmark TNF-α signaling via NF-κB (**F**) and Hallmark IFN-α response gene sets (**G**) in naive Th in HCs, patients with ARB+ lupus, and patients with ARB^–^ lupus. Error is reported as SD. ATAC-Seq data represent 46 naive Th samples (31 patients with lupus + 1 duplicated second time point sample in **A**, 14 healthy in **B** and **C**). RNA-Seq data represent 36 samples (24 lupus, 12 healthy) (**D**–**G**). Across the 24 patients with lupus presented, 9 are prescribed ARBs and 15 are not (**F** and **G**). *P < 0.05, **P < 0.01, ***P < 0.001, multiple 1-way ANOVA with Tukey’s multiple comparisons correction (**C**–**G**).

We found that randomly selected patients with lupus separated into 2 groups with distinct Th chromatin accessibility. However, questions remained regarding the longevity of chromatin states among patients with lupus and upstream mechanisms linked to changes in signaling and subsequent chromatin accessibility. To test whether the absence of disease-associated chromatin in group 2 was stable, we recalled 1 participant at a second time point. The 2 samples from this group 2 patient with lupus clustered together, indicating large-scale maintenance of chromatin architecture over a 2-year period (Figure 6A and Supplemental Figure 7, A and B).

We then looked further into the clinical backgrounds of the lupus patients to identify potential upstream corollaries of the epigenetic footprint. Neither age, race, sex, disease-modifying antirheumatic drug usage at the time of accrual, the presence or absence of nephritis, nor the SLEDAI distinguished group 1 and group 2 (Supplemental Table 2). Additionally, participants with a diagnosis of mixed connective tissue disease or Sjögren’s disease were present in both groups (Supplemental Data 8). One group 2 individual had a history of cancer treatment and chemotherapy that might have altered the epigenetic profile (Supplemental Data 8). Instead, we found differences in therapies: 3 group 2 lupus participants were prescribed losartan, an ARB, and 2 were treated with rituximab. ARBs are commonly used in lupus to treat hypertension and reduce proteinuria to protect renal function (57). ARBs have been reported to reduce TNF-α concentrations in those taking them (58) and influence NF-κB–dependent signaling (59). Given this, we hypothesized that ARBs were a potential upstream signal associated with T cell chromatin states in our data.

We expanded the cohort again, including participants in our repository receiving ARBs or rituximab at the time of sample collection, patients prescribed neither, and additional HCs. Hierarchical clustering of ATAC-Seq data again identified several patients with lupus whose epigenetic profiles intermixed with healthy individuals (Figure 6A). Using previously defined lupus DARs, we performed GSVA enrichment analysis as before. Two additional participants fell within group 2, lacking the lupus-associated epigenetic signature (Figure 6B and Supplemental Figure 7A). This second cohort of nonrandomly selected patients and controls showed similar variation of enrichment, and HCs lacked lupus enrichment as expected (Supplemental Figure 7B). There was no relationship between age, sex, race, or the SLEDAI and presence of the lupus epigenetic signature (Supplemental Figure 7C and Supplemental Data 8). Instead, we verified that having a prescription for an ARB correlated with decreased enrichment for lupus-associated chromatin changes (Figure 6C) and accounted for almost all the participants in group 2. This does not seem to be due to alterations in the renin-angiotensin pathway, as prescription of angiotensin-converting enzyme inhibitors had no effect on patient groupings (Supplemental Figure 7D). Several patients prescribed rituximab lacked the lupus epigenetic signature (Supplemental Figure 7E). However, these patients were uniformly prescribed ARBs, suggesting that ARB use is the dominant factor influencing epigenetic state (Supplemental Figure 7E). ARB use did not correlate with the SLEDAI in this cross-sectional study (Supplemental Figure 7F).

In this extended cohort, the transcriptomes of group 1 and group 2 participants remained distinguished by differences in TNF-α signaling transcriptional enrichment (Figure 6D), with no significant differences in either the IFN-α or the IFN-γ response gene sets (Figure 6E and Supplemental Figure 7, G and H). In parallel with the epigenetic landscape, patients with lupus prescribed ARBs lacked TNF-α signaling enrichment. Analyses of transcriptional networks in autoimmunity have suggested that TNF-α and type I IFNs oppose each other molecularly. It is thus interesting that patients prescribed ARBs lack TNF-α signaling enrichment and have greater IFN-α response enrichment, and the opposite is true of patients not prescribed ARBs (Figure 6, F and G).

### TNF family cytokines are altered in patients with lupus on ARBs and correlate with epigenetic changes in naive Th cells.

**Figure 7:**
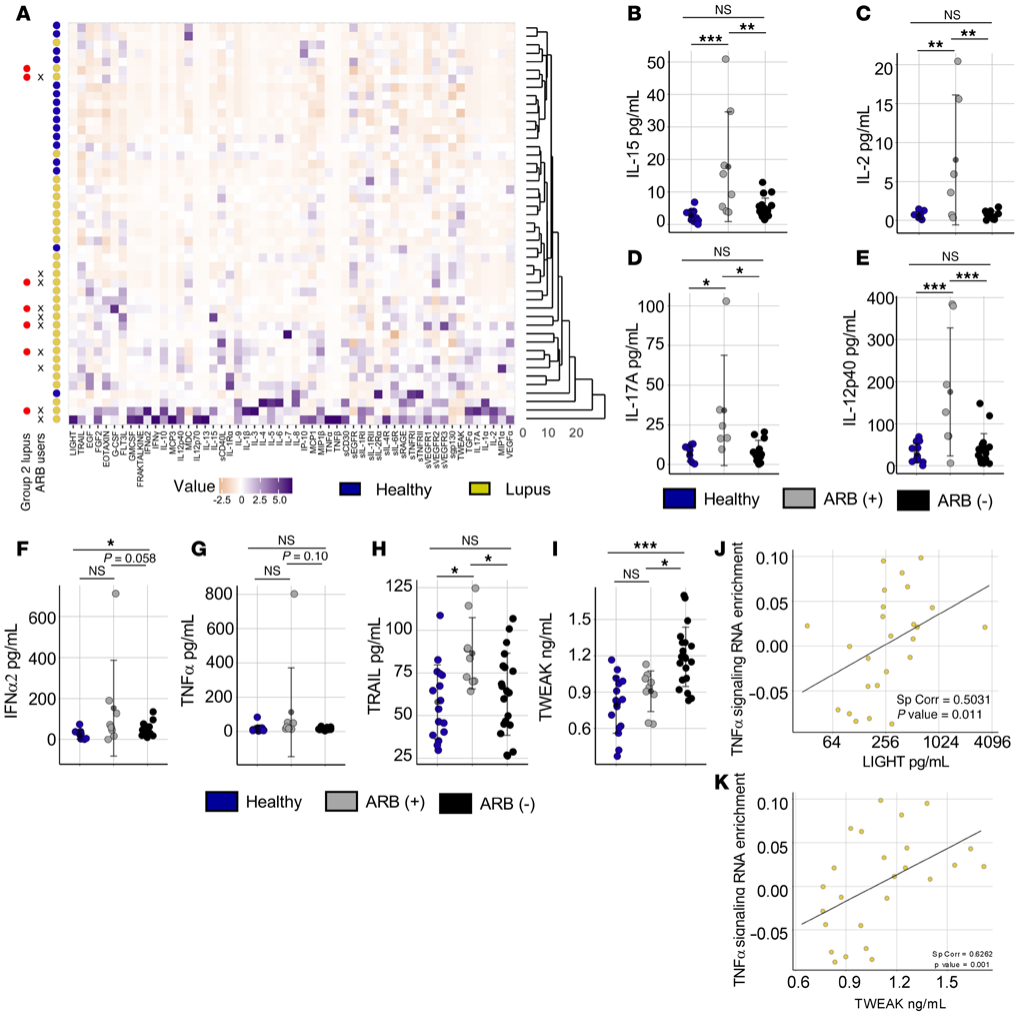
TNF family cytokines correlate with ARB use and epigenetic dysregulation of lupus Th cells. (**A**) Heatmap of plasma analytes (*x* axis) in hierarchically clustered lupus and HCs. Group 2 sorted patients with lupus and ARB-prescribed patients with lupus are noted. (**B**–**I**) Plasma concentrations of selected analytes in HCs (*n* = 16), patients with lupus prescribed ARBs (*n* = 9), and patients with lupus not prescribed ARBs (*n* = 21). Analytes shown are IL-15 (**B**), IL-2 (**C**), IL-17A (**D**), IL-12p40 (**E**), IFN-α2 (**F**), TNF-α (**G**), TRAIL (**H**), and TWEAK (**I**). (**J**) Scatterplot showing linear regression line and Spearman’s correlation coefficient measuring the relationship between plasma LIGHT concentrations (*x* axis) and RNA-Seq GSVA enrichment scores for Hallmark TNF-α signaling via NF-κB in naive Th for concordant lupus samples (*n* = 25). (**K**) Scatterplot showing linear regression line and Spearman’s correlation coefficient measuring the relationship between plasma TWEAK concentrations (*x* axis) and RNA-Seq GSVA enrichment scores for Hallmark TNF-α signaling via NF-κB in naive Th for concordant lupus samples (*n* = 25). Error is reported as SD. *P < 0.05, **P < 0.01, ***P < 0.001, multiple 1-way ANOVA with Tukey’s multiple comparisons correction (**B**–**I**).

Our data clearly identified an association between TNF-α signaling and an altered T cell epigenetic landscape (Figure 5D and Figure 6D). We further demonstrated an association between the use of ARBs and the lupus T cell epigenetic state (Figure 6C). It is possible that direct inhibition of angiotensin II receptor type 1 on T cells by ARB therapy alters molecular state, but low to undetectable *AGTR1* expression in naive T cells suggests that this is not the case (Supplemental Figure 8A). Instead, changes in cytokine abundance in patients on ARBs might mediate epigenetic and transcriptional effects indirectly. To test this, we profiled select plasma cytokines (Figure 7). There was significant variability among lupus participants; however, lupus participants segregated in hierarchical clustering of cytokines with a trend toward increasing proinflammatory profiles (Figure 7A). Several lupus participants had increased levels of IFN-α2, IFN-γ, and IL-1β, as have been previously described (Figure 7A) (41, 60).

Because our data suggested that ARB use correlated with changes in transcriptional TNF-α signaling and to epigenetic state, we focused on the effects of ARBs on cytokine profiles. Patients prescribed ARBs had increased levels of IL-15, IL-2, IL-17A, IL-12p40, IL-10, and sIL-1R1 (Figure 7, B–E, and Supplemental Figure 8, B and C). Lupus nephritis with proteinuria is a frequent indication for ARB prescription and has long been associated with overproduction of IL-17 and abundant Th17 cells (61). In contrast, ARB treatment of mice promotes expansion of Tregs, which produce IL-10 (62, 63). IFN-α2 was similarly increased among several patients on ARBs (Figure 7F), potentially reflecting transcriptional data showing enrichment for IFN-α responses in patients prescribed ARBs (Figure 6G).

TNF-α concentrations did not differ in patients with lupus separated by ARB prescription (Figure 7G). In contrast, other TNF family members, including TRAIL and TWEAK, were altered in association with ARB prescription (Figure 7, H and I). TRAIL was elevated in patients prescribed ARBs while TWEAK was the only cytokine we measured that was decreased in lupus participants prescribed ARBs. TWEAK was also significantly higher in group 1 patients with lupus compared with HCs while group 2 patients with lupus did not have altered TWEAK levels (Supplemental Figure 8D). TWEAK was likewise strongly correlated to the transcriptional activation of TNF-α signaling in naive Th cells of patients with lupus (Figure 7K). Levels of LIGHT also significantly correlated with T cell TNF-α signaling enrichment across patients with lupus (Figure 7J). These strong correlations between epigenetic changes and TNF family member cytokine abundance implicate them in the development of the lupus epigenetic landscape and its regulation by ARBs.

## Discussion

Here, we provide a detailed analysis of chromatin accessibility in naive and Tfh lineage Th cells in lupus and HCs. Naive and effector Th cells in most patients with lupus have enhanced chromatin accessibility with parallel transcriptional changes surrounding cytokine signaling pathway genes, including the TNF signaling family. Single-cell multiome analysis showed that changes to accessibility in lupus naive Th cells are ubiquitous in all subpopulations and not driven by heterogeneity of the compartment. The chromatin landscape of a subset of patients with lupus prescribed ARBs resembles that of HCs. The use of ARBs is also linked to altered cytokine profiles, including reduced TWEAK, and TWEAK levels directly correlate with T cell TNF-α signaling enrichment. These findings have important implications for how we consider disease-associated cytokine production, treat and study different lupus patient subgroups, evaluate naive Th cell responses, and use ARBs in lupus treatment.

Dysregulation of Th cell functions and altered frequencies of cell subsets in lupus might be the result of disease-driven Th cells with abnormal phenotypes and lost cellular identity. For example, others have linked CXCR5^+^CD4^+^ circulating T cell populations to autoantibody production and disease activity (12, 13, 64). However, our data suggest that the chromatin accessibility specific to Th subsets is maintained in lupus and that cellular identity is not widely affected. Nonetheless, lupus Th cells demonstrated robust changes to their chromatin accessibility profiles compared with healthy cells, which might have an influence on their function. In these participants, cTfh and AcTfh cells maintain their subset-driven chromatin accessibility, but lupus AcTfh cells more closely resemble GC Tfh cells than their healthy counterparts. Inappropriate activation of Tfh and altered function in lupus may be linked to cytokine-associated signaling and changes to the epigenome defined here.

Questions of marker fidelity and cell identity are particularly challenging in naive Th cells. Because many studies have previously used only CD45RA positivity when evaluating lupus naive Th populations (17, 18, 20), it is possible that memory Th cells with reactivated CD45RA expression might contaminate isolated cells. Our restrictive gating using CD45RA^+^CD27^+^CD4^+^ naive cells in bulk accessibility assays resulted in chromatin accessibility profiles that maintained published naive chromatin accessibility signatures but were nonetheless affected by disease-associated changes. Single-cell multiome ATAC- and nuclear RNA-Seq of CD45RA^+^CD27^+^CD4^+^ T cells supported this finding, and we identified several previously defined subpopulations within naive Th cells. In lupus, the distribution and cell architecture of these subpopulations were maintained, and instead, we demonstrated that the lupus epigenetic signature penetrated all naive subpopulations, including true naive Th cells. It would be of great interest to examine whether these changes to naive and effector Th biology influence the many observed functional deficiencies and alterations of lupus Th cells during activation and differentiation.

Lupus is heterogeneous with significant variability in transcriptional phenotypes, inflammatory signatures, cytokine expression, and disease activity. These differences have been used to group patients with lupus into meaningful categories, which inform how we think of the disease (40, 41, 65–67). Our characterization utilized the epigenetic landscape. We identified 2 groups of patients with lupus, independent of disease activity and demographics, distinguished by the presence (group 1) or absence (group 2) of lupus-specific Th chromatin accessibility. This separation allowed for extended clinical analysis and better understanding of the potential mechanisms influencing chromatin changes.

Transcriptionally, TNF-α signaling was highly enriched in lupus Th populations along with other cytokine pathways compared with healthy cells. However, when we directly compared the epigenetically distinct groups of patients with lupus, only group 1 lupus individuals with disease-altered chromatin were enriched for TNF-α signaling. Clinical review linked reduced TNF-α signaling enrichment and absence of the lupus chromatin state in group 2 patients to ARB prescription. ARBs are used to treat hypertension and have a beneficial effect on reducing proteinuria and improving renal and cardiovascular outcomes in lupus (68), but they are not generally used as disease-modifying agents. Angiotensin receptors are highly expressed on endothelial cells, and ARB effects on T cells may be indirect. However, multiple immunologic effects of angiotensin inhibition have been documented, including changes in cytokine and chemokine production and Th differentiation. Hypertensive effects of angiotensin depend on TNF-α, and angiotensin inhibition reduced NF-κB signaling and TNF-α production (58, 59, 69, 70). In keeping with these observations, patients with lupus prescribed ARBs were less likely to have the lupus-driven chromatin accessibility signature and had reduced transcriptional enrichment for the TNF-α signaling pathway. Patients prescribed ARBs also had significantly higher plasma IL-10, IL-17, and other cytokines (Figure 7, D–F and H, and Supplemental Figures 8, B and C). These changes in cytokine profiles may relate to disease characteristics prior to ARB prescription or may be a direct effect of ARB. Longitudinal study of patients with lupus initiating ARB therapy would distinguish between these possibilities and indicate whether ARB usage influences cytokines in lupus to alter chromatin and transcriptional states and clinical outcomes.

The heterogeneity in TNF-α signaling, potentially linked to ARB usage, in epigenetically distinct patients is noteworthy given the complex relationship between TNF-α and lupus. TNF-α levels are increased in lupus patient plasma (71), and elevated levels of sTNFR1 and sTNFR2 predate lupus nephritis flares (72). TNF-α blockade in autoimmune diseases like rheumatoid arthritis is associated with the unmasking of a type I IFN signature and the development of drug-induced lupus (73, 74). Studies of both plasmacytoid DCs and macrophages suggest that the 2 cytokines oppose each other (75, 76). This complex interplay between TNF-α and type I IFNs may explain why absence of the epigenetic signature in group 2 participants is not associated with clear differences in disease activity. Heightened type I IFN activity may predate ARB prescription or could be exacerbated by TNF inhibition in patients prescribed ARBs. It is, however, difficult to interpret these results in a cross-sectional study, as we do not know the participants’ SLEDAI or IFN levels prior to ARB prescription. It will be important to design assays of the epigenetic state in individual patients before and after ARB prescriptions and to consider a trial of ARBs in patients without nephritis, the most common indication for the drugs.

The identified TNF transcriptional enrichment in lupus stems from cytokine signaling through classical NF-κB–dependent pathways but may not be directly mediated by TNF-α itself. Multiple TNF family cytokines signal through TRAFs to activate canonical or noncanonical NF-κB signaling cascades (77). LIGHT leads to the activation of NF-κB and AKT signaling associated with T cell proliferation, survival, and effector responses and has been linked to inflammation (78, 79). TWEAK is capable of activating both canonical and noncanonical NF-κB signaling and has previously been linked to the development of lupus nephritis (52, 80, 81). Other TNF cytokines and receptors not specifically evaluated cannot be discounted. At a single time point, TNF-α was not differentially abundant in the plasma of the 2 groups. Instead, patients prescribed ARBs had reduced plasma TWEAK, which significantly correlated with naive Th cell TNF-α signaling enrichment and epigenetic state. T cell signaling through the TWEAK receptor (Fn14; TWEAKR) is not well understood, and mouse T cells may not express TWEAKR (82). We found that only a small percentage of human T cells express TWEAKR, perhaps arguing against a direct signaling mechanism. LIGHT plasma levels were also increased in our lupus cohort, and lupus patients have reduced HVEM expression on T cell surfaces compared with healthy individuals. Similar to TWEAK, LIGHT levels are correlated to the TNF-α transcriptional signature and thus implicated in TNF-α signaling responses in lupus subgroups.

Our data resolve the chromatin accessibility landscape of Th cells in lupus and demonstrate that they acquire disease-associated changes to cytokine-related loci. The ubiquitous nature of chromatin accessibility changes in affected individuals, including in naive Th cells, might suggest a common signaling mechanism, such as a circulating cytokine. Based on our data, we can postulate that TNF family cytokines act on Th cells in some patients with lupus to alter transcriptional and epigenetic features. Introduction of ARBs may indirectly inhibit this TNF phenotype through modulation of TNF family cytokine levels. TNF-α, TWEAK, and LIGHT should all be studied as putative effectors in this process. The stratification of patients with lupus into 2 distinct groups based on the presence of disease-associated chromatin accessibility changes and the use of ARBs provides a unique avenue for study and potential for ARBs beyond their current clinical indication. Finally, the association of TNF signaling with chromatin changes in Th cells in lupus supports further study of TNF family cytokines and how TNF-related chromatin accessibility changes might influence the function of lupus T cells and contribute to, or limit, disease.

## Methods

### Sex as a biological variable.

Because lupus is a disease that disproportionately affects women, most participants included in this study are female.

### Human sample processing.

Patients with lupus were recruited from outpatient visits at the University of Pennsylvania. Venous blood was collected in K2EDTA tubes. PBMCs and plasma samples were isolated using Ficoll-Paque (MilliporeSigma) or Lymphoprep (STEMCELL Technologies) reagents and density gradient centrifugation at 350*g*. HCs were similarly processed. Additional control PBMCs were obtained from the Human Immunology Core at the University of Pennsylvania, where PBMCs were isolated from whole blood or leukapheresis products by similar methods. All PBMCs were cryopreserved in medium containing 10% DMSO.

### Flow cytometry and cell sorting.

For ATAC-Seq and RNA-Seq studies, cryopreserved PBMCs were enriched for Th by negative selection (STEMCELL Technologies 100-0696). Th cells were stained (Supplemental Table 3) and sorted using a BD FACSAria II to isolate naive Th (CD4^+^CD45RA^+^CD27^+^), circulating Tfh (CXCR5^+^PD1^+^CD38^–^ICOS^–^), activated circulating Tfh (CXCR5^+^PD1^+^CD38^+^ICOS^+^), and Th1 cells (CXCR5^–^PD1^–^CXCR3^+^).

Cryopreserved PBMCs from patients with lupus and HCs were thawed, washed, and stained (Supplemental Table 4) for separate flow cytometry phenotyping.

### RNA-Seq.

Cells were processed for RNA-Seq following manufacturer protocols (Takara SMART-Seq v4 Ultra Low Input RNA Kit). Libraries were sequenced by the Center for Spatial and Functional Genomics at the Children’s Hospital of Philadelphia using NovaSeq 6000 paired-end sequencing. FASTQ files were pseudo-aligned to hg38 with Kallisto (83), and count data were imported into R for filtering, quality assessment, and statistics. DEGs (FDR < 0.05) were determined utilizing DESeq2 (84). Sample-wise GSVA enrichment of selected gene sets was calculated using GSVA (85).

### qPCR.

Cryopreserved naive Th cells were thawed and RNA was isolated (QIAGEN RNeasy Mini Kit). cDNA was prepared (Thermo Fisher Scientific 4368814). TaqMan PCR probes for KLF6 (Thermo Fisher Scientific Assay Hs00154550_m1), IFI44 (Thermo Fisher Scientific Assay Hs00197427_m1), IFI27 (Thermo Fisher Scientific Assay Hs01086373_g1), RELB (Thermo Fisher Scientific Assay Hs00232399_m1), CD83 (Thermo Fisher Scientific Assay Hs00188486_m1), and SDHA (Thermo Fisher Scientific Assay Hs00417200_m1) were used for quantification. Reported 2^ΔΔCt^ values were calculated using SDHA as a control gene.

### ATAC-Seq.

Cells were processed for ATAC-Seq according to standard protocols (31). Libraries were sequenced as with the RNA-Seq: NovaSeq 6000 system paired-end sequencing. ATAC-Seq data were analyzed and processed using the ENCODE ATAC-Seq pipeline (https://github.com/ENCODE-DCC/atac-Seq-pipeline; commit ID 47ba8df). FASTQ files were aligned to hg38 using Bowtie2. Duplicates and blacklist regions were filtered, and open chromatin peaks were called with MACS2 (FDR 0.01). A combined peak list was made containing all Th samples using bedtools. Normalization and DAR analysis were performed using DESeq2. Genomic and gene annotations were performed using ChIPSeeker, and enrichment analyses were done with ChipEnrich and contained gene sets belonging to the GO Biological Processes and Hallmark gene set collections (35, 37, 86, 87). Sample-wise enrichment of chromatin data for selected gene sets or selected chromatin region sets was performed utilizing GSVA (85). Transcription factor motif analyses were performed using HOMER (88). Taiji TF analysis and network analysis was performed as described (42).

### Published chromatin accessibility data.

Chromatin accessibility peaks enriched in GC Tfh relative to other T cells were obtained from publicly available data (NCBI GEO GSE130794) (30). To identify Th chromatin accessibility peaks that define naive Th cells, published data of human Th cells were analyzed (GSE179613 and GSE179593) using PCA (34).

### Plasma analysis.

Magnetic bead–based Milliplex assays (MilliporeSigma) were performed with the help of the Human Immunology Core at the University of Pennsylvania. Plasma analyte concentrations were detected on a FLEXMAP 3D instrument running Luminex xPONENT 4.2; Bio-Plex Manager Software 6.1. Analytes measured included immune-targeted assays (HCYTMAG-60K-PX38 and HSCRMAG-32K-PX14 kits) and researcher-selected analytes LIGHT (HCVD1MAG-67K-01), TWEAK (HCMBMAG-22k-01), and TRAIL (HCYP2MAG-62K-01).

### SLE GWAS analysis.

Published GWAS-defined lupus SNPs were linked to proxy SNPs with a linkage disequilibrium *r*^2^ cutoff of 0.5 as described (8, 36).

### 10x Genomics single-cell sequencing.

Cryopreserved PBMCs were thawed prior to negative selection. At least 100,000 naive Th cells (CD45RA^+^CD27^+^) were sorted from 3 healthy and 6 lupus participants. All procedures were done according to 10x Genomics protocols (10x Genomics 1000285, 10x Genomics 2000264, 10x Genomics 2000261). Libraries were quantified and assessed for quality by TapeStation (Agilent 2200 TapeStation system) before sequencing; 10x libraries were sequenced on a NextSeq2000 platform. Chromium (10x Genomics) procedures and sequencing were performed in collaboration with the Center for Host Microbial Interactions at the University of Pennsylvania School of Veterinary Medicine.

Preprocessing of 10x multiome ATAC-Seq and GEX data were performed using Cell Ranger (10x Genomics) and human assembly hg38. Data were loaded into R and filtered for enhanced quality control (43). RNA count data and ATAC count data were independently normalized and integrated, then merged. Normalization and subsequent dimensionality reduction by weighted nearest neighbor analysis were done using Signac (44). Annotation and functional analyses of identified chromatin regions, including sample-wise enrichment analyses, were performed as previously described for bulk-sequenced populations.

### Statistics.

DESeq2 was used to identify DEGs and DARs from sequencing data obtained from analyses of bulk cell populations. FDR less than 0.05 was used throughout the study, and ATAC-Seq data were restricted to FDR < 0.025 and absolute log_2_ FC greater than 0.58 for further analyses (Figure 2). Enrichment scores of selected gene sets and peak sets were calculated as above using the normalized difference in empirical cumulative distribution functions of gene/region ranks inside and outside the gene set or chromatin region set (85). Where applicable, an unpaired, 2-sided *t* test, with Welch’s correction, was applied for direct hypothesis testing. Multiple 1-way ANOVAs with multiple comparisons correction (Tukey’s) were performed as needed. Data are graphed with mean ± SD overlaid. For correlative analyses, a linear correlation along a scatterplot was drawn, and Spearman’s correlation coefficients are reported (Figure 7). *P* < 0.05 was considered statistically significant.

### Study approval.

All human participant research was performed after receipt of written informed consent in accordance with and with the approval of the institutional review boards at the University of Pennsylvania.

### Data availability.

RNA-Seq, ATAC-Seq, and 10x Genomics multiome sequences are available for download at the National Center for Biotechnology Information Gene Expression Omnibus (GSE287248, GSE287250, GSE287249). Raw data for graphs are found in the Supporting Data Values.

## Author contributions

TML conceived the original idea, supervised the research, acquired funding, and contributed edits to the manuscript. APH and TML designed the study with guidance from ADW and DPB. ALK, SWS, JSD, and TML acquired patient samples. SWS and JSD performed clinical evaluation and SLEDAI of recruited participants. JJK performed secondary clinical review and masked analyses. JFB consulted on statistical applications. APH performed the research and data analysis and created visualizations. APH drafted the first version of the manuscript.

## Supplementary Material

Supplemental data

Supplemental data set 1

Supplemental data set 2

Supplemental data set 3

Supplemental data set 4

Supplemental data set 5

Supplemental data set 6

Supplemental data set 7

Supplemental data set 8

Supporting data values

## Version 1 12/17/2024

In-Press Preview

## Version 2 02/10/2025

Electronic publication
