# Supplementary material for: Angiotensin receptor blockers modulate the lupus CD4+ T cell epigenome characterized by TNF family–linked signaling
Source: JCI Insight. 2024 Dec 17;10(3):e176811. doi: 10.1172/jci.insight.176811 (PMC11948580; doi:10.1172/jci.insight.176811)
Supplement: Supplemental data [file jciinsight-10-176811-s102.pdf]

A

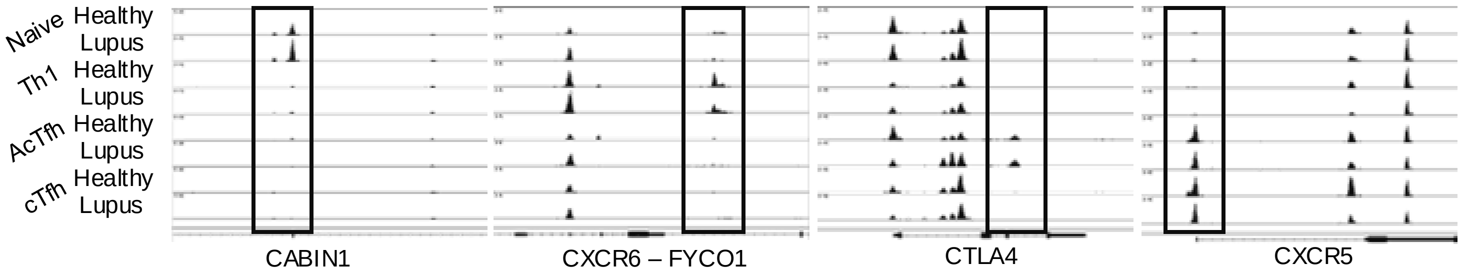

Figure S1: Subset defining chromatin regions are maintained in lupus CD4⁺ T cells. A) Representative chromatin accessibility tracks of healthy (n=1) and lupus (n=1) naïve CD4⁺ T cells, Th1, AcTfh, and cTfh. Selected loci were chosen from subset-defining principal components and include *CABIN1*, *CXCR6*, *CTLA4*, and *CXCR5*.

A

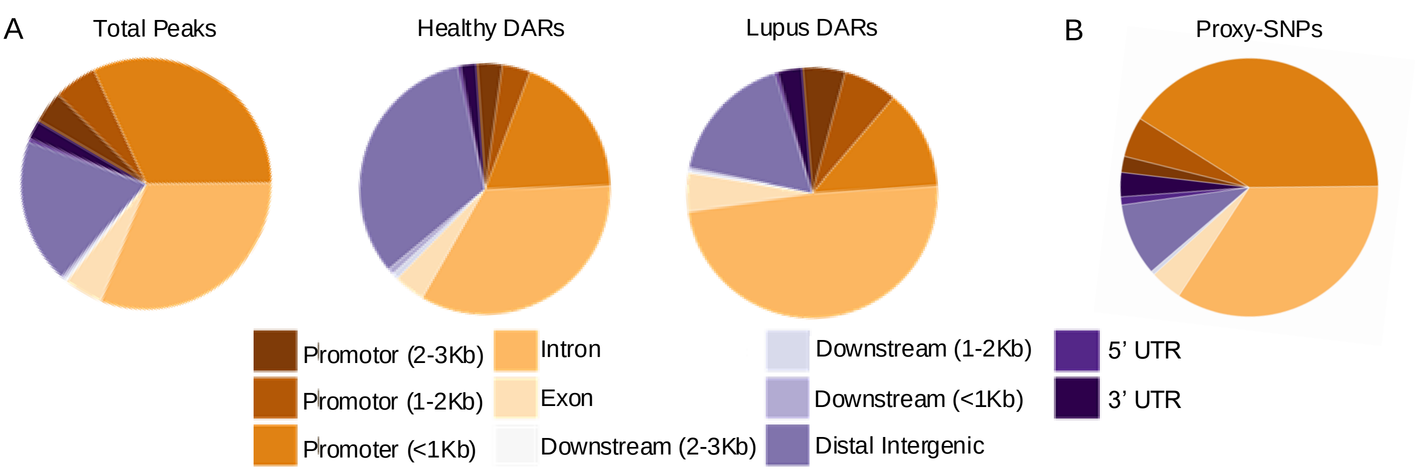

C

	Motif.Name	Consensus	P.value	Log.P.value
1	Fosl2(bZIP)/3T3L1-Fosl2-ChIP-Seq(GSE56872)/Homer	NATGASTCABNN	1e-79	-183.30
2	Jun-AP1(bZIP)/K562-cJun-ChIP-Seq(GSE31477)/Homer	GATGASTCATCN	1e-79	-183.10
3	Fra2(bZIP)/Striatum-Fra2-ChIP-Seq(GSE43429)/Homer	GGATGACTCATC	1e-73	-168.50
4	JunB(bZIP)/DendriticCells-Junb-ChIP-Seq(GSE36099)/Homer	RATGASTCAT	1e-72	-166.90
5	Fra1(bZIP)/BT549-Fra1-ChIP-Seq(GSE46166)/Homer	NNATGASTCATH	1e-71	-164.10
6	Fos(bZIP)/TSC-Fos-ChIP-Seq(GSE110950)/Homer	NDATGASTCAYN	1e-70	-162.90
7	BATF(bZIP)/Th17-BATF-ChIP-Seq(GSE39756)/Homer	DATGASTCAT	1e-65	-150.60
8	Atf3(bZIP)/GBM-ATF3-ChIP-Seq(GSE33912)/Homer	DATGASTCATHN	1e-64	-147.70
9	NFkB-p65(RHD)/GM12787-p65-ChIP-Seq(GSE19485)/Homer	WGGGGATTTCCC	1e-61	-140.70
10	AP-1(bZIP)/ThioMac-PU.1-ChIP-Seq(GSE21512)/Homer	VTGACTCATC	1e-55	-126.90
11	NFkB-p65-Rel(RHD)/ThioMac-LPS-Expression(GSE23622)/Homer	GGAAATTCCC	1e-40	-92.61
12	Bach2(bZIP)/OCILy7-Bach2-ChIP-Seq(GSE44420)/Homer	TGCTGAGTCA	1e-36	-84.84
13	NFkB-p50,p52(RHD)/Monocyte-p50-ChIP-Chip(Schreiber_et_al.)/Homer	GGGGGAATCCCC	1e-23	-55.25
14	Rfx2(HTH)/LoVo-RFX2-ChIP-Seq(GSE49402)/Homer	GTTGCCATGGCAACM	1e-17	-39.94
15	RFX(HTH)/K562-RFX3-ChIP-Seq(SRA012198)/Homer	CGGTTGCCATGGCAAC	1e-15	-35.32

D

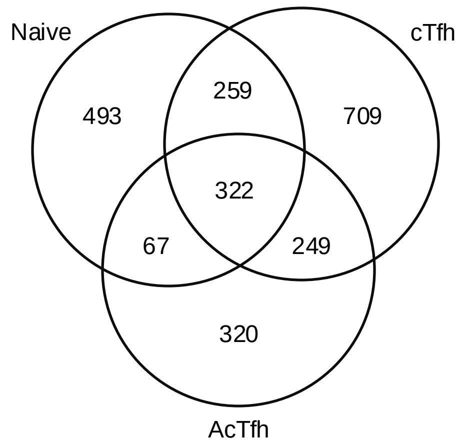

E

Cell Subset	Peak	FDR	Gene Annotation
AcTfh	Chr2:203960595-203961922	0.0029144	ICOS
Naive	Chr2:203998274-203998704	0.0065310	ICOS
cTfh	Chr2:204004952-204006813	0.0249306	ICOS
cTfh	Chr2:204016046-204016271	0.0108942	ICOS

Figure S2: DARs in lupus CD4⁺ T cells are enriched for intronic regions. A) Accessible chromatin regions from combined cell subsets (Naïve, TH1, AcTfh, and cTfh)(left), DARs with greater accessibility in healthy CD4⁺ T cells (middle) and DARs with greater accessibility in lupus (right) were annotated relative to gene position. Frequency of promoter, exon, intron, downstream, and distal intergenic regions are graphed. **B**) 198 Proxy SNPs linked ($r2 > 0.5$) to published lupus GWAS susceptibility SNPs and which overlap with open chromatin regions were annotated relative to gene position. **C**) HOMER TF motif analysis results for DARs which overlap a gene promoter and are more accessible in lupus CD4⁺ T cell subsets. The top 15 most significant Motifs are shown. **D**) DARs were separately determined for naïve CD4⁺ T cells, AcTfh, and cTfh by comparing lupus and healthy samples using DESEQ2 (FDR <0.025). The overlap of DARs for each subset is shown via Venn diagram to highlight overlap across cell subsets. **E**) Subset specific DARs which are found in naïve, cTfh, or AcTfh, and map to the *ICOS* locus are listed. ATAC data represent 25 naïve CD4⁺ T cell samples (13 Lupus, 12 Healthy), 8 Th1 samples (4 Lupus, 4 Healthy), 24 cTfh samples (12 Lupus, 12 Healthy), and 24 AcTfh samples (12 Lupus, 12 Healthy) (**A-E**).

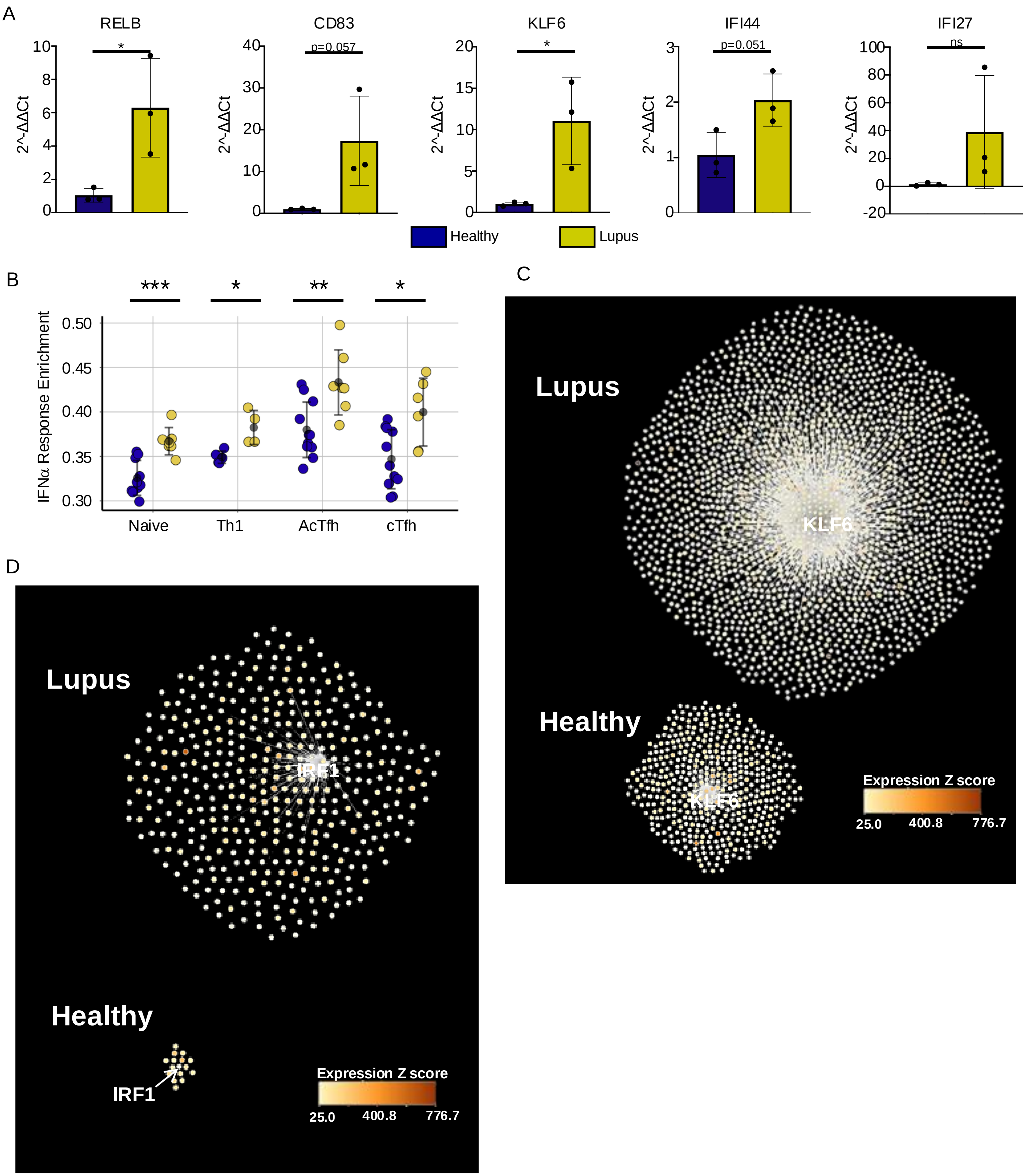
A
RELB
CD83
KLF6
IFI44
IFI27
2^-ΔΔCt
*
p=0.057
*
p=0.051
ns
Healthy
Lupus
B
IFNα Response Enrichment
***
*
**
*
Naive
Th1
AcTfh
cTfh
C
Lupus
KLF6
Healthy
KLF6
Expression Z score
25.0
400.8
776.7
D
Lupus
IRF1
Healthy
IRF1
Expression Z score
25.0
400.8
776.7

Figure S3: TNF- and IFN-related pathways are transcriptionally activated in lupus CD4⁺ T cells. A) qPCR 2^-ΔΔCt values of selected IFNα response genes and TNFα signaling genes. RNA was collected from sorted naïve $CD4^+$ T cells from lupus subjects (n=3) and healthy controls (n=3). **B)** Hallmark IFNα response gene set variation enrichment scores across sorted $CD4^+$ T cell populations in lupus subjects and healthy controls. **C)** Taiji-defined KLF6 TF gene regulatory networks among lupus or healthy naïve $CD4^+$ T cells (edge weight cutoff = 100). Node color saturation is proportional to node expression. **D)** Taiji-defined IRF1 TF gene regulatory networks among lupus or healthy naïve $CD4^+$ T cells (edge weight cutoff = 100). Node color saturation is proportional to node expression. *P < 0.05; **P < 0.01, ***P <0.001 paired 2-tailed t tests (**A-B**). RNA-seq data represent 17 naïve $CD4^+$ T cell samples (7 Lupus, 10 Healthy), 9 Th1 samples (4 Lupus, 5 Healthy), 17 cTfh samples (6 Lupus, 11 Healthy), and 18 AcTfh samples (7 Lupus, 11 Healthy) **(B)**.

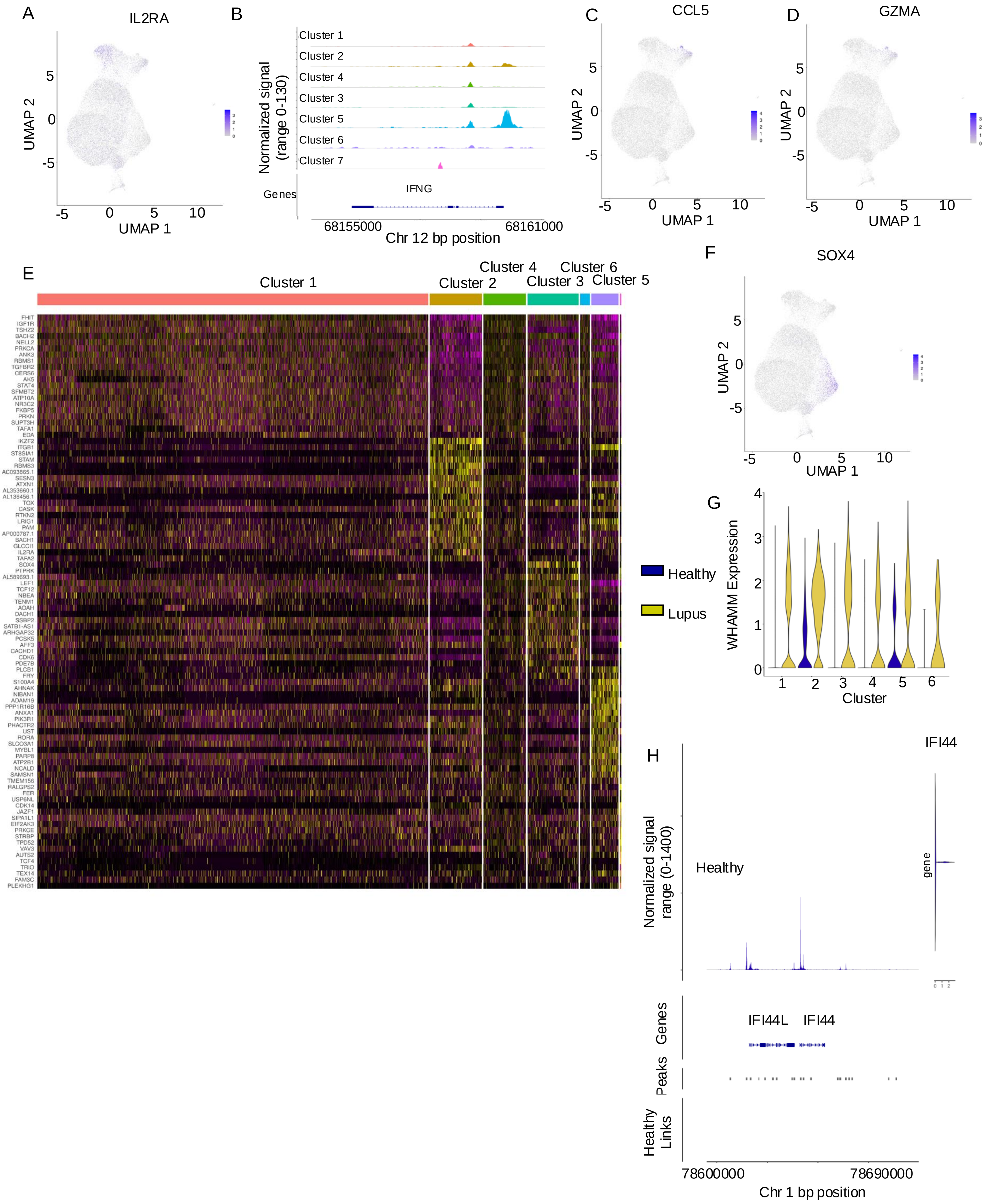
A
IL2RA
UMAP 2
UMAP 1
B
Normalized signal (range 0-130)
Cluster 1
Cluster 2
Cluster 4
Cluster 3
Cluster 5
Cluster 6
Cluster 7
Genes
IFNG
68155000
68161000
Chr 12 bp position
C
CCL5
D
GZMA
E
Cluster 1
Cluster 2
Cluster 4
Cluster 3
Cluster 6
Cluster 5
F
SOX4
G
Healthy
Lupus
WHAMM Expression
Cluster
H
IFI44
Healthy
Normalized signal range (0-1400)
gene
Genes
IFI44L
IFI44
Peaks
Healthy Links
78600000
78690000
Chr 1 bp position

Figure S4: Multi-omic single cell accessibility and transcription profiles reveal that lupus naive $CD4^+$ T cells maintain normal subpopulation and cell state distributions in disease. A) UMAP feature plot of single cell multi-ome clusters of naïve $CD4^+$ T cells depicting *IL2RA* gene expression. **B**) *IFNG* locus track visualization of relative chromatin accessibility across naïve $CD4^+$ T cell clusters. **C**) UMAP feature plot of single cell multi-ome clusters of naïve $CD4^+$ T cells depicting *CCL5* gene expression. **D**) UMAP feature plot of single cell multi-ome clusters of naïve $CD4^+$ T cells depicting *GZMA* gene expression. **E**) Heatmap of top DEG for each naïve $CD4^+$ T cell cluster. **F**) UMAP feature plot of single cell multi-ome clusters of naïve $CD4^+$ T cells depicting *SOX4* gene expression. **G**) Violin plot depicting relative *WHAMM* gene expression in lupus and healthy cells across naïve $CD4^+$ T cell clusters. **H**) Peak-Gene linkage analysis results depicting chromatin accessibility track (top), gene expression (top right), and peak-gene linkages (bottom) along the IFI44 locus in healthy naïve $CD4^+$ T cells. Single cell multi-ome data include cell from 6 lupus and 3 healthy subjects (**A-H**).

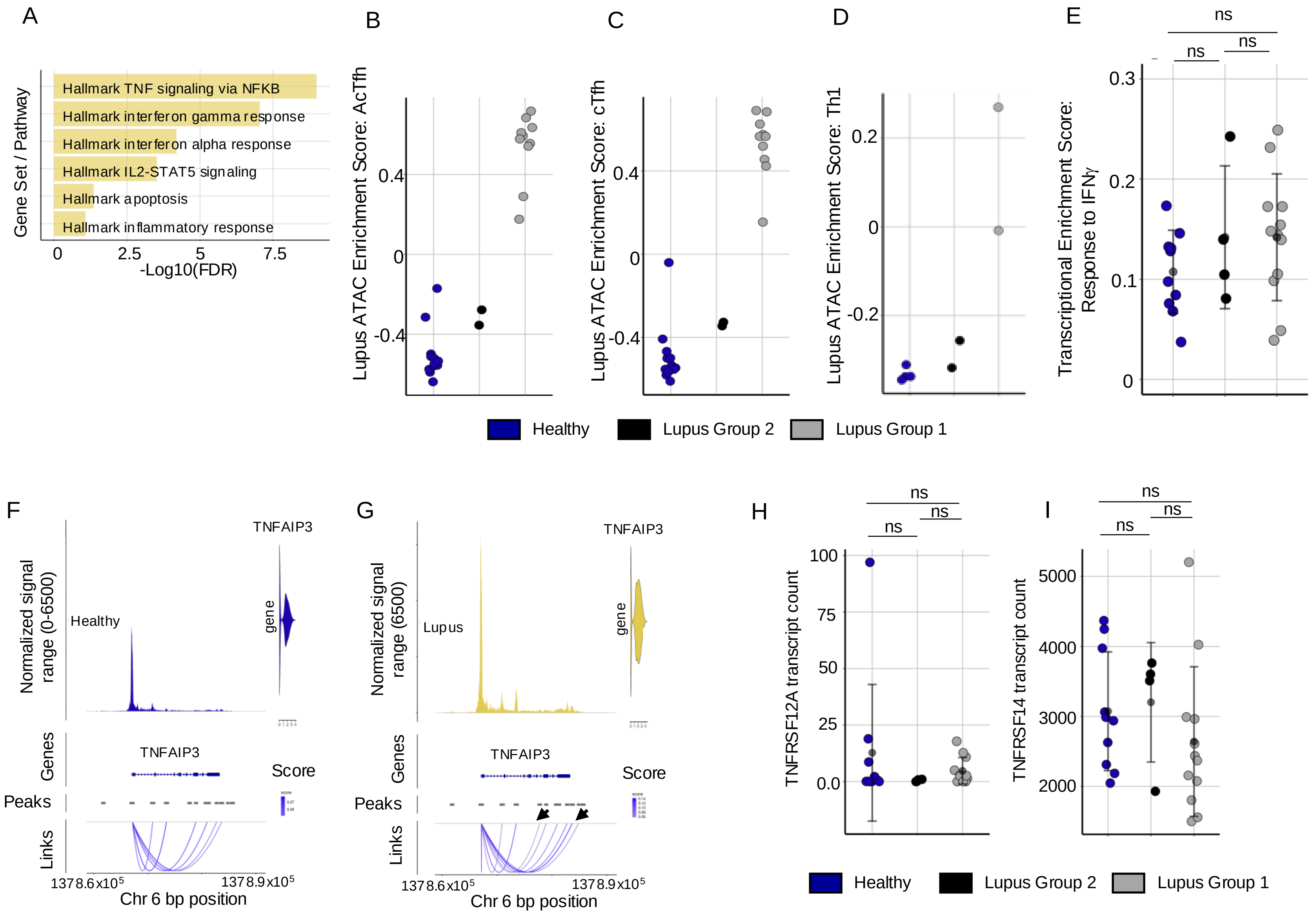

Figure S5: Lupus patients exhibit heterogeneity in chromatin accessibility dysregulation. A) Pathway enrichment analysis results for bulk-isolated ATAC defined DARs in sorted lupus naïve CD4$^+$ T cells. Regions with increased accessibility in lupus cells were used. GO:Biological Process and Hallmark gene sets were used. **B-D**) Lupus signature enrichment (sample-wise peak-set variation enrichment of lupus-associated DARs) (Figure 2C, DARs n= 2683) among AcTfh, cTfh, or Th1 cells. Group 1 lupus patients, Group 2 lupus patients, and healthy controls are shown. **E**) RNAseq-defined Hallmark IFNγ response gene set variation enrichment scores across naïve CD4$^+$ T cells in Group 1 lupus subjects, Group 2 lupus subjects, and healthy controls. **F-G**) Peak-gene linkages depicting chromatin accessibility track (top), gene expression (top right), and peak-gene linkages (bottom) along the *TNFAIP3* locus in healthy (F) and lupus (G) naïve CD4$^+$ T cells. **H-I**) *TNFRSF12A* (H) and *TNFRSF14* (I) gene transcript counts in Group 1 lupus subjects, Group 2 lupus subjects, and healthy controls. Error is reported as SD. *P < 0.05; **P < 0.01, ***P <0.001 multiple one-way ANOVA with Tukey multiple comparisons correction (E, H, I). ATAC data represent 34 Naïve CD4$^+$ T cell samples (22 Lupus, 12 Healthy) **(A)** or ATAC data represent 8 Th1 samples (4 Lupus, 4 Healthy), 24 cTfh samples (12 Lupus, 12 Healthy), and 24 AcTfh samples (12 Lupus, 12 Healthy) (**B-D**). RNA-seq data represent 25 samples (15 Lupus, 10 Healthy) (**E, H, I**). 10X single cell multi-ome data include cells from 6 lupus and 3 healthy subjects (F-G).

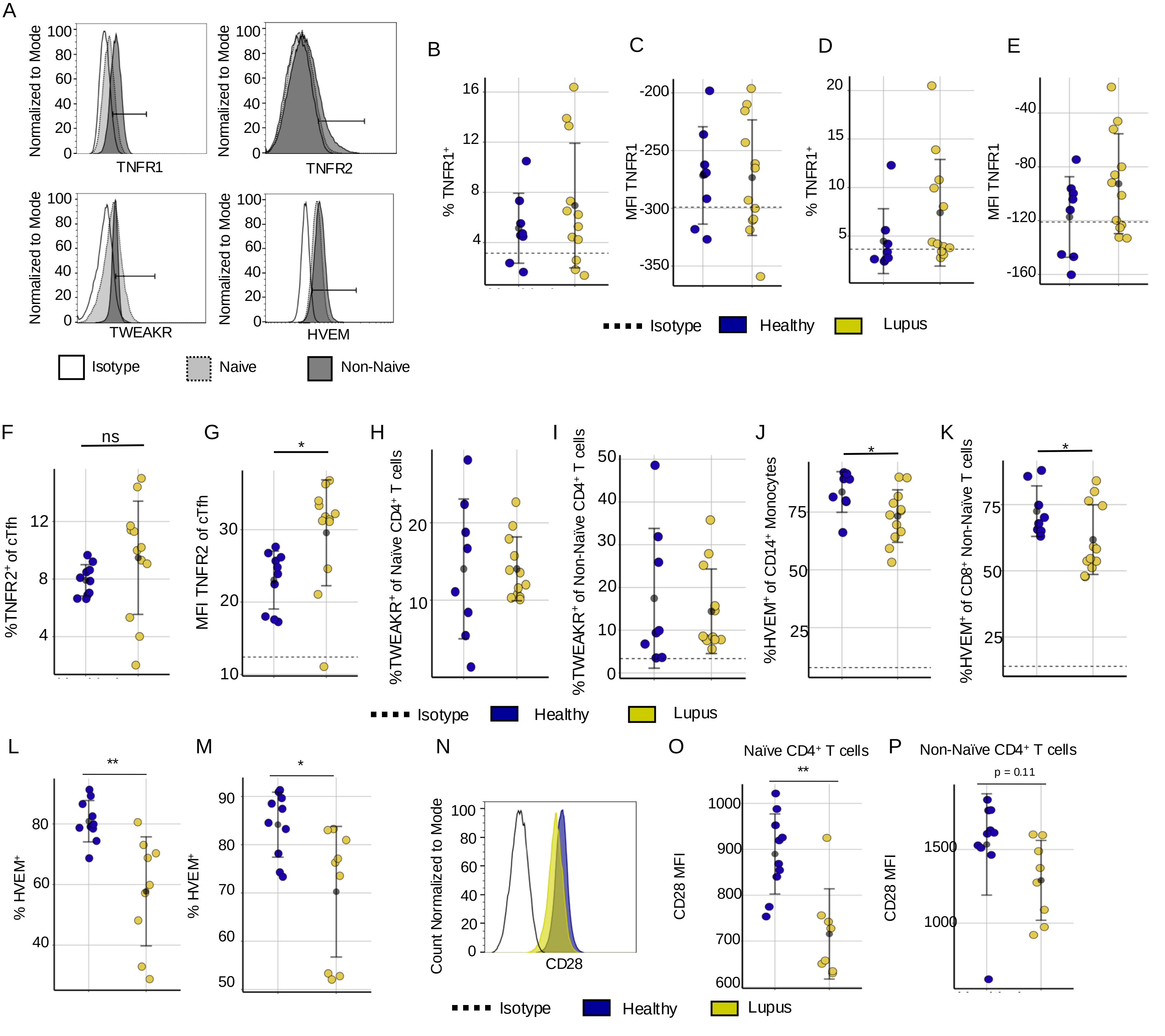

Figure S6: Protein-level dysregulation of TNFRs suggests active TNF signaling in lupus T cells. A) Representative flow cytometry histograms showing positive gating of TNFR1, TNFR2, TWEAKR (fn14), and HVEM in naïve ($CD45RA^+CD27^+$) and non-naïve ($CD45RA^+CD27^-$, $CD45RA^-CD27^-$, $CD45RA^-CD27^+$) $CD4^+$ T cells. **B-C**) Frequency of $TNFR1^+$ (B) and TNFR1 MFI (C) amongst naïve $CD4^+$ T cells in lupus patients (n= 12) and healthy controls (n=8). **D-E**) Frequency of $TNFR1^+$ (D) and TNFR1 MFI (E) amongst non-naïve $CD4^+$ T cells in lupus patients and healthy controls (left). **F-G**) Frequency of $TNFR2^+$ cells among cTfh $CD4^+$ T cells in lupus patients (n = 12) and healthy controls (n = 10) (F); TNFR2 MFI (G). **H-I**) Frequency of $TWEAKR^+$ naïve (H) and non-naïve $CD4^+$ T cells (I) in lupus patients (n = 12) and healthy controls (n = 8). **J**) Frequency of $HVEM^+$ of $CD14^+$ monocytes in lupus patients (n = 12) and healthy controls (n = 8). **K**) Frequency of $HVEM^+$ non-naïve $CD8^+$ T cells in lupus patients (n = 12) and healthy controls (n = 8). **L-M**) Frequency of HVEM+ cells among naïve CD4+ T cells (L) and non-naïve CD4+ T cells (M) in Group 1 lupus patients (n = 8) and healthy controls (n= 10). **N**) Representative flow cytometry histograms showing CD28 expression in naive and non-naïve CD4+ T cells. **O-P**) CD28 MFI in naïve (O) and non-naïve (P) CD4+ T cells of Group 1 lupus patients (n = 8) and healthy controls (n=10).Error reported as SD. $^*P < 0.05$; $^{**}P < 0.01$, $^{***}P < 0.001$ determined using 2-tailed t tests (B-P).

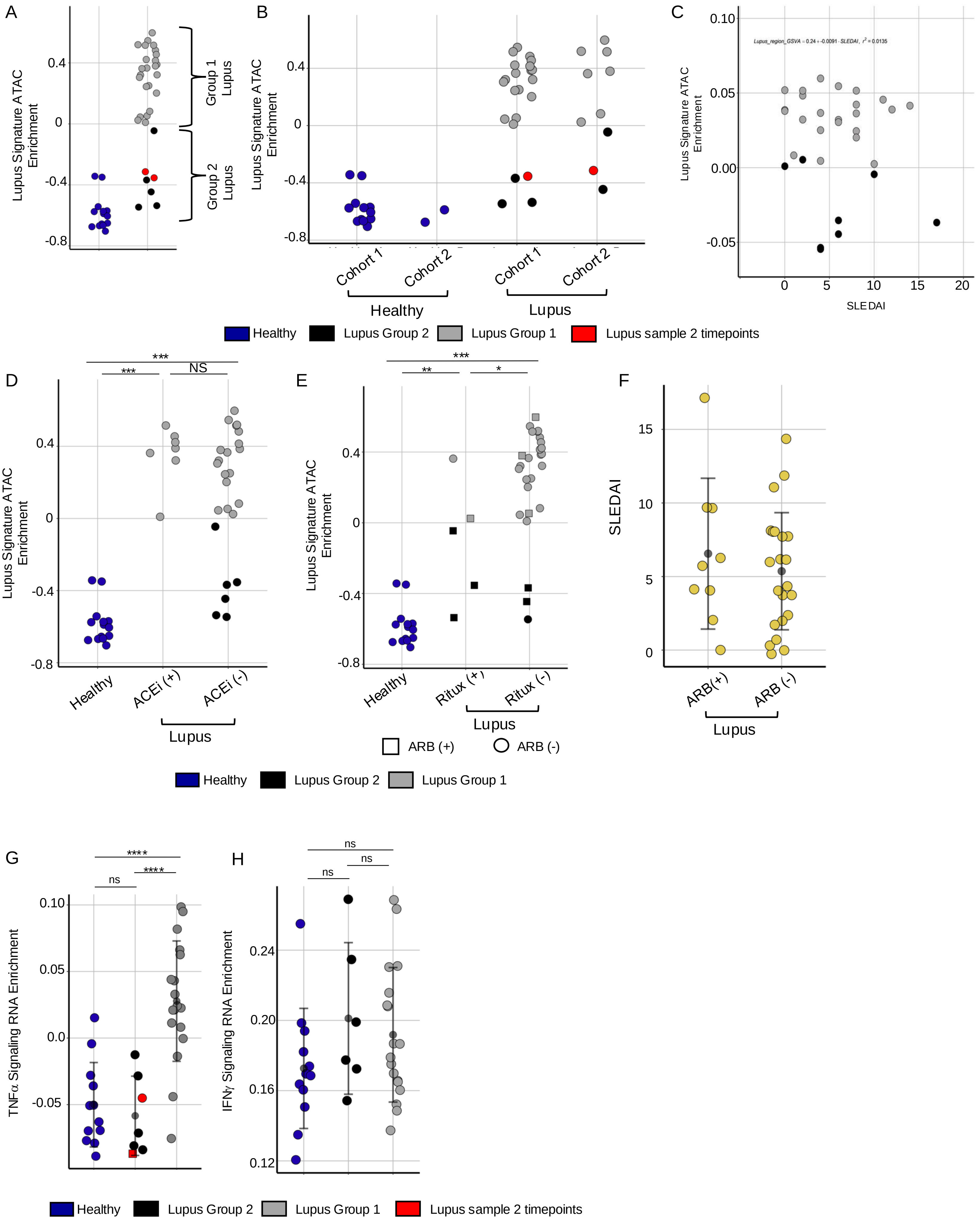

A
Lupus Signature ATAC Enrichment
0.4
0
-0.4
-0.8
Group 1 Lupus
Group 2 Lupus
B
Lupus Signature ATAC Enrichment
0.4
0
-0.4
-0.8
Cohort 1
Cohort 2
Cohort 1
Cohort 2
Healthy
Lupus
C
Lupus Signature ATAC Enrichment
0.10
0.05
0.00
-0.05
Lupus_region_GSVA = 0.24 + -0.0091 · SLEDAI, r² = 0.0135
0
5
10
15
20
SLEDAI
Healthy
Lupus Group 2
Lupus Group 1
Lupus sample 2 timepoints
D
***
***
NS
Lupus Signature ATAC Enrichment
0.4
0
-0.4
-0.8
Healthy
ACEi (+)
ACEi (-)
Lupus
E
***
**
*
Lupus Signature ATAC Enrichment
0.4
0
-0.4
-0.8
Healthy
Ritux (+)
Ritux (-)
Lupus
ARB (+)
ARB (-)
F
SLEDAI
15
10
5
0
ARB(+)
ARB (-)
Lupus
Healthy
Lupus Group 2
Lupus Group 1
G
****
****
ns
TNFα Signaling RNA Enrichment
0.10
0.05
0.0
-0.05
H
ns
ns
ns
IFNγ Signaling RNA Enrichment
0.24
0.20
0.16
0.12
Healthy
Lupus Group 2
Lupus Group 1
Lupus sample 2 timepoints

Figure S7: ACEi prescription and SLEDAI are not associated with epigenetic and transcriptional changes in lupus T cells. A) Enrichment of signature lupus-associated DARs (Figure 2C, n= 2683) among naïve $CD4^+$ T cell samples in this expanded dataset. Highlighted (red) is a cross-timepoint replicate spanning 2 years and indicating epigenetic phenotype stability. **B**) Enrichment of signature lupus-associated DARs (Figure 2C, n= 2683) in naïve $CD4^+$ T cells graphed in healthy individuals (blue) and lupus individuals separated by randomly selected (cohort1) and targeted selection (cohort 2) cohorts. **C**) Enrichment of signature lupus-associated DARs (Figure 2C, n= 2683) in naïve $CD4^+$ T cells graphed against lupus patient SLEDAI. **D)** Enrichment of signature lupus-associated DARs (Figure 2C, n= 2683) in naïve $CD4^+$ T cells graphed in healthy individuals and lupus individuals prescribed (ACEi +) and not prescribed (ACE -) angiotensin converting enzyme inhibitors. **E**) Enrichment of signature lupus-associated DARs (Figure 2C, n= 2683) in naïve $CD4^+$ T cells graphed in healthy individuals and lupus individuals prescribed (Ritux +) and not prescribed (Ritux -) Rituximab. Lupus patients prescribed ARBs are denoted as squares. Lupus patients not prescribed ARBs are denoted as circles. **F**) SLEDAI in ARB-prescribed lupus subjects and those not prescribed ARBs. **G**) RNA-seq data GSVA of Hallmark TNFα signaling via NFκB in naïve $CD4^+$ T cells in Group 1 lupus subjects, Group 2 lupus subjects, and healthy controls. Highlighted (red) is a cross-timepoint replicate spanning 2 year. **H**) RNA-seq data GSVA of Hallmark IFNγ signaling gene set genes in naïve $CD4^+$ T cells in Group 1 lupus subjects, Group 2 lupus subjects, and healthy controls. Error is reported as SD. ATAC data represent 45 unique naïve $CD4^+$ T cell samples (31 Lupus patients and 14 Healthy subjects) (A-F). RNA-seq data represent 36 samples (24 Lupus, 12 Healthy) (G-H). An additional lupus sample representing a second time point of a previously profiled patient appears in panels A, B, and G. *$P < 0.05$; **$P < 0.01$, ***$P < 0.001$ multiple one-way ANOVA with Tukey multiple comparisons correction (D-E, G-H).

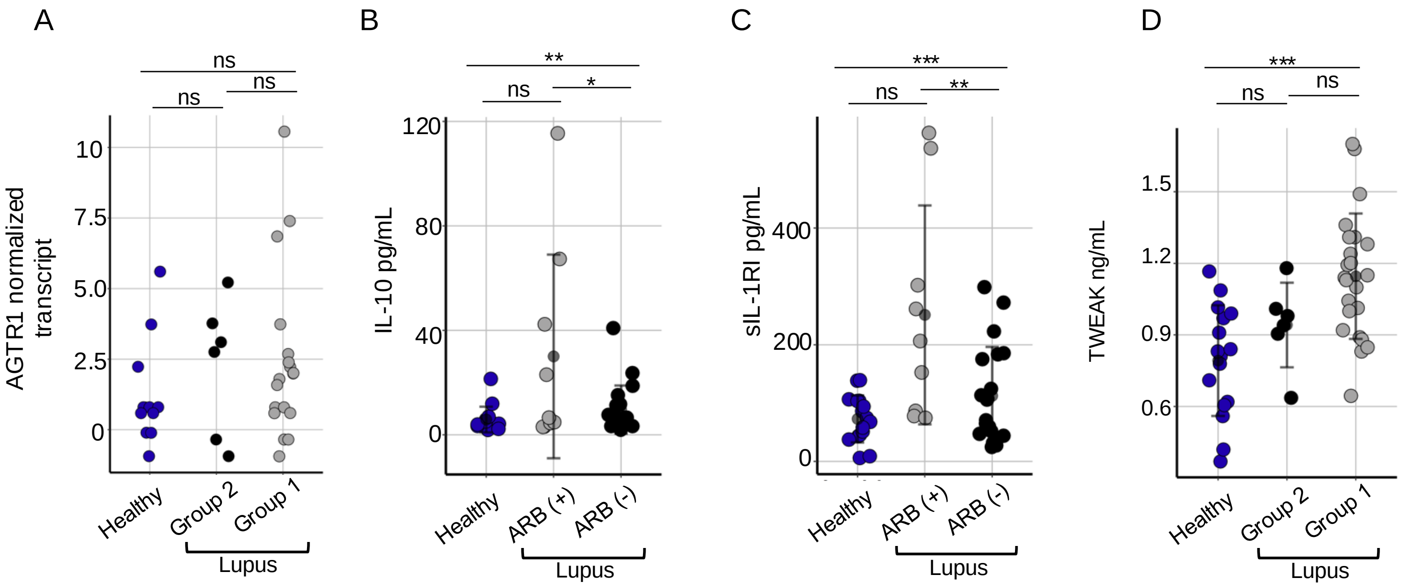

Figure S8: Lupus patient plasma contains soluble mediators of inflammation and activation. **A)** Normalized RNA transcript levels of AGTR1 in healthy patients (n = 12), Group 2 lupus patients (n = 6), and Group 1 lupus patients (n = 18). **B-C**) Plasma concentrations of selected analytes in healthy controls (n = 16), lupus patients prescribed ARBs (n= 9), and lupus patients not prescribed ARBs (n=21). Analytes shown are IL-10 (B) and sIL-1R1 (C). **D**). Plasma concentrations of TWEAK in healthy controls (n = 16), Group 2 lupus patients (n= 6), and Group 1 lupus patients(n=24). Error is reported as SD. *P < 0.05; **P < 0.01, ***P <0.001 multiple one-way ANOVA with Tukey multiple comparisons correction (A-D).

Table S1	Patient sample data Figures 1-3	
	**Healthy**	**Lupus**
**Subjects represented (n)**	16	13
**Age (median)**	40	38
**Age (range)**	25-53	22-56
**Female (ratio)**	13/16	12/13
**Race**	UNK	Asian(1), Black(7), White(5)
**AcTfh (n)**	12 (ATAC)<br>11 (RNA)	12 (ATAC)<br>7 (RNA)
**cTfh (n)**	12 (ATAC)<br>11 (RNA)	12 (ATAC)<br>6 (RNA)
**Naive CD4 (n)**	12 (ATAC)<br>10 (RNA)	13 (ATAC)<br>7 (RNA)
**Th1 (n)**	4 (ATAC)<br>5 (RNA)	4 (ATAC)<br>4 (RNA)
**SLEDAI (median)**		4
**SLEDAI (range)**		0-12
**Corticosteroid**		13 (ever) 6 (active)
**Mycophenolate mofetil**		8 (ever) 2 (active)
**Azathioprine**		5 (ever) 3 (active)
**Methotrexate**		4 (ever) 1 (active)
**Cyclophosphamide**		2 (ever) 0 (active)
**Rituximab**		1 (ever) 0 (active)
**Belimumab**		3 (ever) 1 (active)
**NSAIDs**		10 (ever) 7 (active)
**Hydroxychloroquine**		13 (ever) 10 (active)

Supplementary Table 1: Subject demographics. Descriptions of subjects and demographics for samples appearing in Figures 1-3.

Table S2	ATAC/RNA Sample Data Fig. 5		
	**Healthy**	**Group 1 Lupus**	**Group 2 Lupus**
**Subjects represented (n)**	14	18	4
**Age (median)**	41	37.5	41
**Age (range)**	25-53	22-68	31-71
**Female (ratio)**	11/14	17/18	4/4
**Race**	NA	Asian(2), Black(9), White(7)	Asian(0), Black(2), White(2)
**Naive CD4 (n)**	12 (ATAC) 10 (RNA)	18 (ATAC) 11 (RNA)	4 (ATAC) 4 (RNA)
**SLEDAI (median)**		5	5
**SLEDAI (range)**		0-14	4-17
**Corticosteroid**		18 (ever) 9 (active)	4 (ever) 2 (active)
**Mycophenolate mofetil**		11 (ever) 4 (active)	2 (ever) 2 (active)
**Azathioprine**		6 (ever) 3 (active)	2 (ever) 1 (active)
**Methotrexate**		7 (ever) 3 (active)	0 (ever) 0 (active)
**Cyclophosphamide**		3 (ever) 0 (active)	0 (ever) 0 (active)
**Rituximab**		1 (ever) 0 (active)	2 (ever) 0 (active)
**Belimumab**		4 (ever) 1 (active)	2 (ever) 0 (active)
**NSAIDs**		16 (ever) 10 (active)	4 (ever) 3 (active)
**Hydroxychloroquine**		18 (ever) 12 (active)	4 (ever) 3 (active)

Supplementary Table 2: Subject demographics. Descriptions of subjects and demographics for samples appearing in Figure 5.

Table S3: Antibodies used in cell sorting					
Antibody	Clone	Channel	Company	Catalog	Figure
CXCR5	RF8B2	APC-R700	BDbiosciences	565191	Figure 1-7
ICOS	C398.4A	BV785	Biolegend	313534	Figure 1-7
CD4	SK3	BV605	BD Biosciences	565998	Figure 1-7
PD-1	EH12.1	BV421	BD Biosciences	562516	Figure 1-7
CD38	HIT2	BB515	BDbiosciences	564499	Figure 1-7
GHOST			Tonbo Biosciences	13-0870-T100	Figure 1-7
CD8a	RPA-T8	APC-cy7	Biolegend	301015	Figure 1-7
CD19	SJ25C1	APC-cy7	Biolegend	363009	Figure 1-7
CD11c	Bu15	APC-cy7	Biolegend	337217	Figure 1-7
CD27	M-T271	PE-Cy7	Biolegend	356411	Figure 1-7
CD45RA	HI100	PE	Biolegend	304107	Figure 1-7
CD14	HCD14	APC-cy7	Biolegend	325619	Figure 1-7
FC shield			BD Biosciences	564219	Figure 1-7

Table S4: Figure 5 & Figure S6 Antibodies					
Antibody	Clone	Channel	Company	Catalog	Figure
CD27	L-128	BUV395	BDbiosciences	563816	Figure 5 & Figure S6
CD3	UCHT1	BUV496	BDbiosciences	612941	Figure 5 & Figure S6
CD45RA	HI100	BUV563	BDbiosciences	612927	Figure 5 & Figure S6
CD20	2H7	BUV805	BDbiosciences	612906	Figure 5 & Figure S6
PD-1	EH12	BV480	BDbiosciences	566175	Figure 5 & Figure S6
FC block			BDbiosciences	564219	Figure 5 & Figure S6
CD19	SJ25C1	BB700	BDbiosciences	566397	Figure 5 & Figure S6
CD38	HIT2	BB515	BDbiosciences	564499	Figure 5 & Figure S6
CXCR5	RF8B2	APC-R700	BDbiosciences	565191	Figure 5 & Figure S6
CD127	HIL-7R-M21	BV650	Bdbiosciences	563225	Figure 5 & Figure S6
CD28	CD28.2	BV605	Bdbiosciences	562976	Figure 5 & Figure S6
CD270 (HVEM)	CW10	BUV661	BDbiosciences	750104	Figure 5 & Figure S6
CCR6	11A9	BB700	BDbiosciences	566477	Figure 5 & Figure S6
CXCR3	1C6/CXCR3	PE cf594	Bdbiosciences	562451	Figure 5 & Figure S6
KI67	11F6	BV421	Biolegend	151208	Figure 5 & Figure S6
CD4	SK3	BV750	Biolegend	344643	Figure 5 & Figure S6
CD8	RPA-T8	BV570	Biolegend	301037	Figure 5 & Figure S6
CXCR3	G025H7	BV711	Biolegend	353731	Figure 5 & Figure S6
ICOS	C398.4A	BV785	Biolegend	313533	Figure 5 & Figure S6
DR5	DJR2-4	PE	Biolegend	307405	Figure 5 & Figure S6
CD57	HNK-1	PE dazzle 594	Biolegend	359619	Figure 5 & Figure S6
CD11c	3.9	APC-fire 750	Biolegend	301645	Figure 5 & Figure S6
CD120b	3G7A02	PE/CY7	Biolegend	358411	Figure 5 & Figure S6
FOXP3	PCH101	PE-CY5.5	ThermoScientific	35-4776-41	Figure 5 & Figure S6
HLA-DR	L2W3	BV605	Biolegend	307640	Figure 5 & Figure S6
CD14	M5E2	BV650	Biolegend	301835	Figure 5 & Figure S6
CD4	SK3	BV750	Biolegend	344643	Figure 5 & Figure S6
CD56	5.1H11	BV785	Biolegend	362549	Figure 5 & Figure S6
HVEM (CD270)	CW10	BB700	BDbiosciences	746034	Figure 5 & Figure S6
fn14	ITEM-4	PE	BDbiosciences	565731	Figure 5 & Figure S6
KI67	B56	PEcf594	BDbiosciences	567120	Figure 5 & Figure S6
BTLA (CD272)	MIH26	Pe-cy7	Biolegend	344516	Figure 5 & Figure S6
CD8a	RPA-T8	APC-cy7	Biolegend	301015	Figure 5 & Figure S6
CD19	SJ25C1	APC-cy7	Biolegend	363009	Figure 5 & Figure S6
TNFR1 (CD120a)	W15099A	APC	Biolegend	369905	Figure 5 & Figure S6
